# Adipose Tissue Immunomodulation: A Novel Therapeutic Approach in Cardiovascular and Metabolic Diseases

**DOI:** 10.3389/fcvm.2020.602088

**Published:** 2020-11-17

**Authors:** Ibrahim AlZaim, Safaa H. Hammoud, Houssam Al-Koussa, Alaa Ghazi, Ali H. Eid, Ahmed F. El-Yazbi

**Affiliations:** ^1^Department of Pharmacology and Toxicology, American University of Beirut, Beirut, Lebanon; ^2^Department of Biochemistry and Molecular Genetics, American University of Beirut, Beirut, Lebanon; ^3^Department of Pharmacology and Therapeutics, Beirut Arab University, Beirut, Lebanon; ^4^Department of Pharmacology and Therapeutics, Faculty of Medicine, American University of Beirut, Beirut, Lebanon; ^5^Department of Basic Medical Sciences, College of Medicine, Qatar University, Doha, Qatar; ^6^Biomedical and Pharmaceutical Research Unit, QU Health, Qatar University, Doha, Qatar; ^7^Department of Pharmacology and Toxicology, Faculty of Pharmacy, Alexandria University, Alexandria, Egypt

**Keywords:** adipose tissue, adipose tissue inflammation-definition of metabolic syndrome-insulin resistance-myokines-systemic inflammation, immunometabolism, adipose tissue immunology, adipose tissue browning

## Abstract

Adipose tissue is a critical regulator of systemic metabolism and bodily homeostasis as it secretes a myriad of adipokines, including inflammatory and anti-inflammatory cytokines. As the main storage pool of lipids, subcutaneous and visceral adipose tissues undergo marked hypertrophy and hyperplasia in response to nutritional excess leading to hypoxia, adipokine dysregulation, and subsequent low-grade inflammation that is characterized by increased infiltration and activation of innate and adaptive immune cells. The specific localization, physiology, susceptibility to inflammation and the heterogeneity of the inflammatory cell population of each adipose depot are unique and thus dictate the possible complications of adipose tissue chronic inflammation. Several lines of evidence link visceral and particularly perivascular, pericardial, and perirenal adipose tissue inflammation to the development of metabolic syndrome, insulin resistance, type 2 diabetes and cardiovascular diseases. In addition to the implication of the immune system in the regulation of adipose tissue function, adipose tissue immune components are pivotal in detrimental or otherwise favorable adipose tissue remodeling and thermogenesis. Adipose tissue resident and infiltrating immune cells undergo metabolic and morphological adaptation based on the systemic energy status and thus a better comprehension of the metabolic regulation of immune cells in adipose tissues is pivotal to address complications of chronic adipose tissue inflammation. In this review, we discuss the role of adipose innate and adaptive immune cells across various physiological and pathophysiological states that pertain to the development or progression of cardiovascular diseases associated with metabolic disorders. Understanding such mechanisms allows for the exploitation of the adipose tissue-immune system crosstalk, exploring how the adipose immune system might be targeted as a strategy to treat cardiovascular derangements associated with metabolic dysfunctions.

## Introduction

Over the past two decades, the traditional view of adipose tissue (AT) as a passive store of excess calories evolved to implicate an endocrine role that is particularly pertinent to glucose and lipid homeostasis (1). This endocrine function is the result of a complex interaction between adipocytes and cells of the stromal vascular fraction of AT, which modulate the type of mediators produced in different conditions of health and disease. Importantly, this endocrine role is ascribed to the white adipose tissue (WAT); one of the two major types of AT. While, WAT comprises unilocular adipocytes that specialize in the storage of energy and the regulation of metabolic homeostasis by the production of adipokines, brown AT (BAT) is formed of mitochondria-rich multilocular adipocytes whose main function is energy dissipation through thermogenesis (2). Interestingly, accumulating evidence shows that both endocrine and thermogenic functions are modulated by resident and infiltrating immune cells. In fact, AT harbors a plethora of immune cells belonging to both the innate and adaptive immune systems, which either exert a pro- or an anti-inflammatory role depending on the microenvironmental stimulation and metabolic rewiring. Obese AT represents a state of chronic inflammation due to increased adipocyte hypertrophy, hyperplasia and apoptosis accompanied by an alteration in the production of adipokines and inflammatory mediators. This has been linked to the development of insulin resistance (IR), metabolic syndrome (MetS) and type 2 diabetes (T2D) (3). The manifestations of AT inflammation are proposed to alter the phenotype and gene expression profile of adipose immune cells, which was proposed to underlie major comorbidities of obesity including cardiovascular diseases (CVDs) (4).

In this review, we elaborate on the metabolic rewiring of AT-resident and infiltrating immune cells in health and disease and their participation in the inflammatory phenotype of AT relevant to the development of metabolic and cardiovascular disorders. We also touch upon recent evidence implicating AT-resident and infiltrating immune cells in the induction or suppression of AT thermogenesis and its possible outcomes. Finally, we discuss how several interventions immuno-modulate AT function and the exciting future perspectives in the field of AT immunometabolism.

## Obesity, at Inflammation and the Metabolic Syndrome

### AT Inflammation and Remodeling

The incidence of obesity is increasing globally at an alarming rate with a parallel increase in the associated conditions including IR, CVD, and T2D (5, 6). Obesity is considered a chronic inflammatory disease that is linked to metabolic disorders (7). In this context, AT chronic low-grade inflammation and the progressive infiltration of immune cells into the AT contribute to IR (5, 8). The precise triggers of obesity-correlated inflammation are not fully understood. However, it is widely accepted that overnutrition drives a state of hyperinsulinemia, which participates in AT inflammation by inducing adipocyte hypertrophy followed by hypoxia, adipocyte death, lipotoxicity, and altered extracellular matrix (ECM).

WAT is a poorly vascularized tissue that exhibits a further decreased blood supply during AT expansion resulting in hypoxia. This hypoxic atmosphere is stimulated by increased adipocyte dimensions beyond the oxygen diffusing-ability, increased oxygen demand and lack of proper compensatory vascularization (9, 10). Infiltrating immune cells and ECM alterations also contribute to this hypoxic phenotype (11). Indeed, hypoxia induces the release of pro-inflammatory cytokines, chemokines, and angiogenic as well as fibrotic factors from adipocytes, which favor AT inflammation, vasculature remodeling, and AT dysfunction (9, 12). Hypoxia-induced AT dysfunction is characterized by an extensive lipolytic activity and free fatty acids (FAs) release leading to lipotoxicity, which was shown to exacerbate AT inflammation and participate in the pathogenesis of IR by promoting endoplasmic reticulum (ER) stress, adipocyte apoptosis, and inflammation (8, 13, 14). Hypoxia also causes necrosis-like adipocyte death, which initiates inflammation via interacting with macrophages (15). Nevertheless, AT reacts to adipocyte death by initiating a self-limiting wound healing response, which is characterized by intensive infiltration of immune cells, especially macrophages, that encircle dead fat cells, creating histological crown-like structures (CLS) (15). These macrophages generate toxic reactive oxygen species (ROS) and nitric oxide (NO), which further damage neighboring cells and support fibrosis (16). On the other hand, as the injury signal sustains in obesity, the chronic stimulation of myofibroblasts and immune cells causes additional damage, fibrosis, ECM remodeling and eventually AT dysfunction as well as IR (17, 18).

AT low-grade inflammation is driven by the excessive production of inflammatory cytokines such as tumor necrosis factor (TNF)-α and interleukin (IL)-1β, which activate and recruit immune cells to AT, promoting its remodeling and causing an imbalance between homeostatic AT-resident immune cells and infiltrating inflammatory immune cells (19). The latter cells consist of macrophages, neutrophils, mast cells and T and B lymphocytes among others that secrete cytokines promoting the recruitment and polarization of other inflammatory cells in the AT. Moreover, dendritic cells (DCs), macrophages, and B cells induce the expansion of CD4 and CD8 T cells in the AT (20–22). In case of obese AT, macrophages exceed 50% of the immune cell population compared to lean AT (23, 24), and the production of CXCL12, CCR5, and MCP-1 by the AT tends to recruit and activate macrophages, making macrophages the major producers of cytokines in the AT (25–27).

### Differences in Inflammation Susceptibility of Different AT Depots

Various molecular, physiological, and metabolic differences exist among adipose depots (28). Differences found in the microenvironment of WAT depots lead to unequal AT expansion and inflammation susceptibility under metabolic stress. Indeed, BAT is less prone to inflammation in comparison to WAT (29–31). Another good example is the difference in inflammation susceptibility between PVAT and other VAT depots. We have shown that PVAT localized inflammation, which was associated with uncoupling protein 1 (UCP1)-mediated hypoxic preconditioning, occurs in isolation of systemic inflammation in a prediabetic rat model (32). Moreover, EpiCAT has small adipocyte size, high protein content and high rate of FA synthesis compared to other adipose depots making it susceptible to a metabolic profile shift (33). Additionally, during EpiCAT expansion, a quick proinflammatory microenvironment is generated due to the extensive inflammatory immune cell infiltration (34).

### Adipokine Profile Dysregulation

Adipokines, which encompass endocrine and other biologically-active proteins, are released by WAT and function as hormones that regulate insulin sensitivity, energy balance, immune system functions and whole-body homeostasis (35). Metabolically healthy individuals possess a balance between proinflammatory and anti-inflammatory adipokines. This balance shifts in favor of proinflammatory mediators as the AT expands in the course of metabolic syndrome and obesity. This adipokine profile dysregulation has been associated with an increased risk of metabolic dysfunction, T2D and CVDs. Importantly, these adipokines profoundly influence the activation state, differentiation, and proliferation of AT-resident and infiltrating immune cells. Anti-inflammatory adipokines include adiponectin, C1q/TNF-related proteins (CTRPs), omentin, and secreted frizzled-related protein 5 (SFRP5) (36–38). Proinflammatory adipokines include leptin, resistin, chemerin, visfatin, retinol binding protein 4 (RBP4), and lipocalin 2 (LCN2) (35).

#### Adiponectin

Adiponectin is the best-known and most abundant adipokine found in human serum with insulin-sensitizing and cardioprotective actions (39, 40). Adiponectin serum levels decrease in obesity, T2D, and in states of high oxidative stress (41, 42). Total plasma adiponectin levels are also inversely correlated with MI risk (43, 44). Adiponectin-deficient mice exhibit an exacerbated myocardial ischemic injury, and adiponectin supplementation protects the heart against ischemia/reperfusion injury (45, 46). In circulation, adiponectin forms low, intermediate, and high molecular weight complexes where the high molecular weight complex was shown to block NF-κB activation and the production of proinflammatory cytokines (47, 48). Adiponectin exerts its effects by binding to its tissue-specific receptors, AdipoR1 and AdipoR2, which results in the downstream activation of AMPK, Akt-eNOS phosphorylation, and NO production (49–51). Moreover, adiponectin exerts an antioxidant (oxidative and nitrative stress) activity that is AMPK-independent and that is largely mediated via PKA-dependent NF-κB inhibition (52). Importantly, adiponectin modulates the activity of several immune cells in the AT including macrophages (53, 54), eosinophils (55), and mast cells (56). Indeed, profound mechanistic frameworks for this modulation are still lacking and require further investigation.

#### CTRPs

CTRPs are structurally similar, paralogs of adiponectin, with at least 15 isoforms being described to date where they exhibit broadly diverse effects (57, 58). For example, CTRP1 plays an important role in regulating body energy homeostasis and insulin sensitivity (59). Plasma CTRP1 was higher and negatively correlated with insulin resistance in diabetic subjects (60, 61). A recent study highlighted a significant association between increased CTRP1 levels and metabolic syndrome, obesity, T2D and non-alcoholic fatty liver disease (62). It was suggested that CTRP1 improves insulin resistance by reducing the phosphorylation of IRS-1 Ser1101 (61). In line with that, it was shown that elevated concentrations of CTRP1 reduce weight gain and diet-induced insulin resistance (59). Moreover, CTRP1 was shown to enhance glucose uptake through an increased GLUT4 translocation to the plasma membrane and enhanced glycolysis in HFD-fed CTRP1 transgenic mice (63). Moreover, CTRP1 promoted fatty acid oxidation and therefore, CTRP1 seems to perform a defensive catabolic effect in response to nutritional challenges. Interestingly, CTRP1-deficient mice fed a low-fat diet developed insulin resistance and hepatic steatosis (64). At the level of the cardiovascular system, CTRP1 was shown to regulate blood pressure through the induction of vasoconstriction (65). As such, mice overexpressing CTRP1 are hypertensive and hypertensive patients display a higher CTRP1 levels in comparison to healthy individuals (65). Moreover, CTRP1 was demonstrated to limit the extent of ischemia-reperfusion injury in acute myocardial infarction (59). The level of CTRP1 was also significantly increased in CAD patients and was suggested as a superior biomarker for the diagnosis of severity of vessel-lesion in CAD patients (66, 67). Interestingly, CRTP1 levels positively correlated with concentrations of IL-6 and TNF-α in CAD patients (66). In congestive heart failure patients, the levels of CTRP1 in serum and EpiCAT were higher than in controls, which was associated with a worse prognosis (68). Nevertheless, the implication of CTRP1 serum levels alteration on the activity of immune cells in models of metabolic and cardiovascular diseases has not yet been assessed.

CTRP3 (also known as cartducin) regulates adiponectin secretion from adipocytes (69, 70). CTRP3 was also shown to regulate glucose homeostasis (71), to stimulate *in vitro* endothelial cell proliferation and migration (58), and to inhibit TLR4 signaling and cytokine production in LPS- and FFA-stimulated adipocytes and monocytes (58). Importantly, CTRP3 serum level decrease following myocardial infarction and its restoration post-MI attenuates post-ischemic pathological remodeling (72).

Plasma CTRP9 levels are decreased in rodent models of obesity and diabetes (73, 74). Importantly, CTRP9 heterodimerizes with adiponectin and shares AdipoR1 stimulation in cultured cardiomyocytes and endothelial cells (73, 75, 76). CTRP9 promotes eNOS activity and NO production via AdipoR1-mediated activation of AMPK, resulting in endothelium-dependent vasorelaxation of aortic rings (76). Moreover, CTRP9 attenuates inflammation in TNF-α-stimulated endothelial cells via AMPK activation and inhibits inflammatory responses in ox-LDL-stimulated macrophages (77, 78). Indeed, CTRP9-deficient mice are obese and insulin resistant (79). Importantly, several studies demonstrated a cardioprotective effect of CTRP9 (73, 74, 80, 81).

Adipolin (CTRP12) is an insulin-sensitizing adipokine that is abundantly produced by AT and whose expression levels decrease in rodent models of obesity (82, 83). The systemic administration of adipolin ameliorated glucose intolerance and insulin resistance in HFD-fed obese mice (82). Adipolin administration also attenuated macrophage infiltration and proinflammatory genes expression in AT of obese mice (82). Importantly, it was demonstrated that adipolin levels increase in response to hyperinsulinemia induction in healthy lean human subjects or following PPARγ agonism (84). This indicates that adipolin, as a novel anti-inflammatory adipokine, increases in the early stages of the metabolic insult to curb metabolic derangements and these levels are not sustained following prolonged metabolic disease induction. Importantly, adipolin levels were found to be lower in CAD patients compared to healthy controls (85). Moreover, adipolin levels were inversely correlated with HOMA-IR and TNF-α and positively correlated with adiponectin expression levels (85). Another study highlighted that adipolin levels decrease in acute myocardial infarction patients and that these levels are negatively associated with epicardial fat thickness (86). Indeed, adipolin-deficient mice exhibited an exacerbated neointimal thickening following vascular injury which was accompanied by enhanced inflammation and vascular cell proliferation (87). Adipolin-treated LPS-stimulated macrophages *in vitro* exhibited a reduced expression of IL-6 and TNF-α. Moreover, adipolin-deficient MI mice had increased myocardial apoptosis, cardiomyocyte hypertrophy, and perivascular fibrosis at the remote zone of infarct heart through an Akt-dependent mechanism (88). This indicates that adipolin exerts a protective effect against pathological processes of vascular and cardiac remodeling.

The adipokine CTRP6 regulates metabolism and inflammation (89, 90). CTRP6 improves cardiac function and ameliorates ventricular remodeling post-MI (91). CTRP13 was also shown to improve insulin sensitivity and inhibit the inflammation of lipid-loaded hepatocytes (92).

#### Omentin

Omentin is a novel adipokine whose levels decrease in obese subjects and negatively correlate with carotid intima media thickness (93–95). Moreover, omentin expression is negatively associated with the prevalence and the angiographic severity of coronary artery disease (96). Omentin inhibits TNF-α-induced endothelial COX2 expression and induces the activity of eNOS (97). Moreover, omentin enhances isolated aortic rings dilation in mice in an eNOS-dependent manner (98). Omentin systemic delivery also attenuated neointimal thickening and vascular smooth muscle proliferation in an AMPK-dependent mechanism (99). Therefore, omentin functions as an anti-atherogenic and anti-inflammatory adipokine similar to adiponectin and the CTRPs.

#### SFRP5

SFRP5 has anti-inflammatory effects in AT and in macrophages where it was shown to suppress the noncanonical Wnt5a/JNK signaling which inhibits the synthesis of macrophage TNF-α, IL-1β, and CCL2-MCP1 (100).

#### Leptin

First described as a satiety hormone, leptin was shown to bind to long form of leptin receptor expressed in nearly all immune cells to initiate innate immune responses (101). Leptin enhances the production of proinflammatory cytokines in peripheral blood monocytes and tissue-resident macrophages in mice and humans (102–105). Leptin also induces ROS production in macrophages, neutrophils, and endothelial cells and potentiate the expression of INFγ-induced nitric oxide synthase (106–108). Leptin also enhances Th1 and Th17 immune responses and prevents T cell apoptosis (109).

#### Resistin

Resistin was first characterized as a mediator of insulin resistance, metabolic syndrome, and T2D in mice (110). Although WAT represents the primary source of resistin in mice, monocytes and macrophages are the most important source of resistin in humans (111). The proinflammatory actions of resistin are mediated by CAP-1, a resistin receptor, with downstream activation of NF-κB in human monocytes (112). Resistin binds to TLR4 and regulates the production of TNF-α and IL-6 in macrophages through the activation of NF-κB signaling (113). Importantly, resistin levels are elevated in obese humans and are associated with an increased risk of CVDs (114).

#### Visfatin

Visfatin, also known as pre-B cell colony-enhancing factor (PBEF), is a novel, highly conserved adipokine that is abundantly expressed in visceral fat (115, 116). Visfatin plays a determinant role in the pathophysiology of metabolic and cardiovascular diseases (117). Visfatin elicits insulomimetic effects in adipocytes and an increased blood glucose level prompts an increase of serum visfatin (115, 118). Nevertheless, it was suggested that the effects of visfatin do not involve the classical insulin signaling pathways in skeletal muscles (115, 119). Indeed, several studies demonstrated an association between increased plasma visfatin level and diabetes (120, 121). In contrast, other studies reported opposite or no association between visfatin plasma levels and diabetes (122, 123). Similar controversy was also documented when correlating visfatin plasma levels with obesity (124–126). Despite the role of visfatin in metabolic disorders remaining debatable (127), it does not rule out visfatin implication in these disorders and its participation in metabolic dysfunction-associated cardiovascular diseases. Several studies suggested a pro-inflammatory role of visfatin in both VAT and scWAT (128). In fact, visfatin was shown to enhance monocyte-mediated recruitment of T cells and B cells by increasing the expression of CD80, CD40, and ICAM-1 (129). Moreover, visfatin-stimulated human leukocytes exhibit a dose-dependent induction in the expression of IL-1β, IL-1Ra, IL-10, and IL-6 (129).

#### LCN2 and RBP4

LCN2, also known as neutrophil gelatinase-associated lipocalin (NGAL) is upregulated in the presence of IFN-γ and TNF-α in obese individuals (130, 131). Similarly, RBP4, which is mostly complexed with retinol in circulation, was shown to promote IR and increases the risk of T2D (132, 133). RBP4 activates antigen presenting cells and is suggested as a cardiometabolic marker in MetS (134).

**Figure 1:**
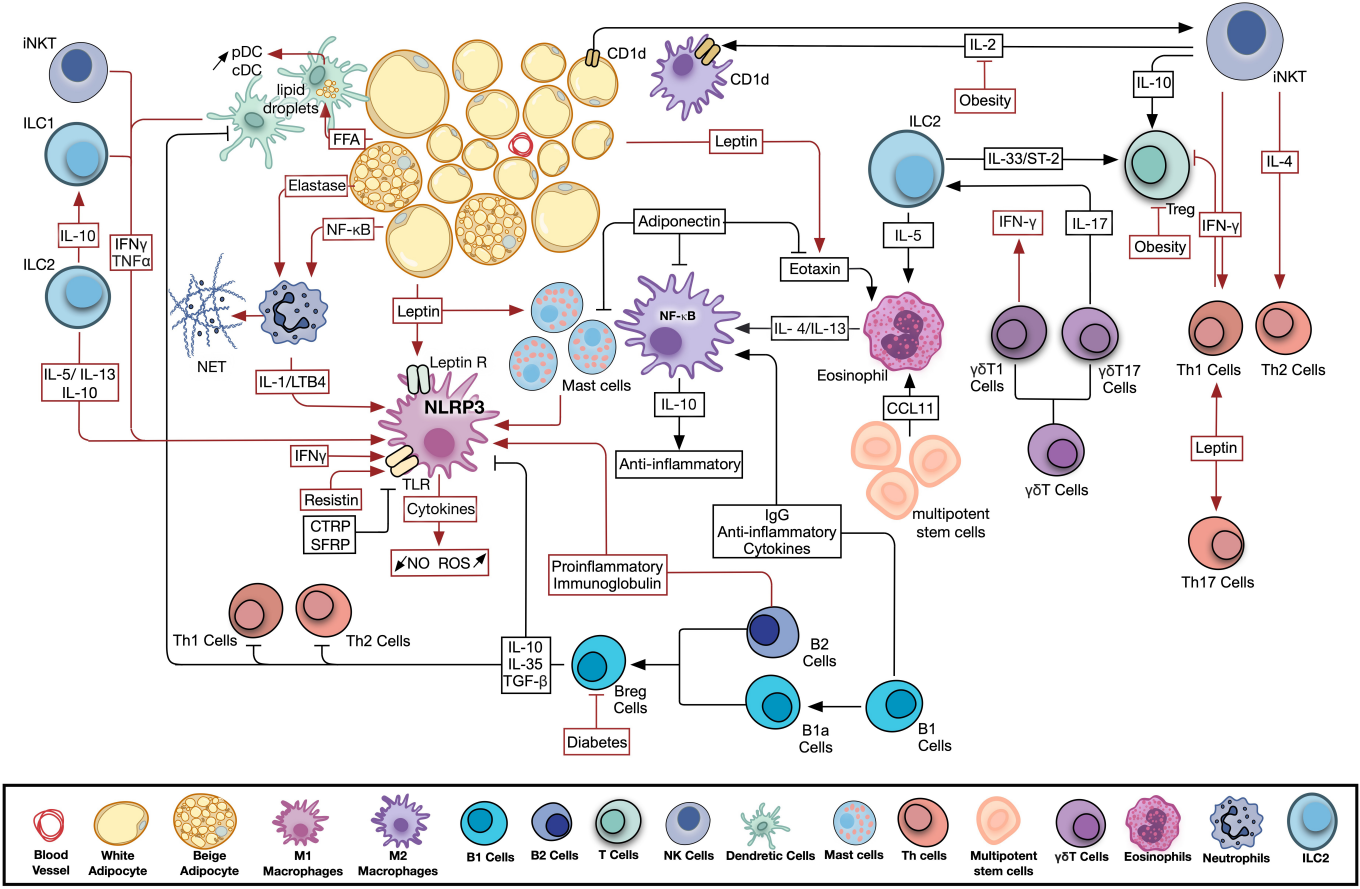
Adipose tissue resident and infiltrating immune cells activity in health and disease. Significant crosstalk exists among adipocytes, adipokines, and resident and infiltrating, innate and adaptive immune cells. Metabolic disease conditions modulate the adipokine profile and immune cell activity leading to the observed chronic low-grade inflammation. Pathways involved in AT homeostasis are depicted in black arrows, while those activated by metabolic dysfunction are shown in red. B Cell, B Lymphocyte; Breg Cells, Regulatory B Lymphocyte; CCL11, C-C motif chemokine 11; CD1d, Cluster of Differentiation 1d; cDC, Conventional Dendritic Cell; FFA, Free Fatty Acids; IFNγ, Interferon Gamma; IgG, Immunoglobulin G; IL, Interleukin; ILC, Innate Lymphoid Cell; iNKT Cell, Invariant Natural Killer T Cell; LTB4, Leukotriene B4; NET, Neutrophil Extracellular Trap; NF-κB, Nuclear Factor Kapp-light-chain-enhancer of Activated B cells; NLRP3, NLR Family Pyrin Domain Containing 3; NO, Nitric Oxide; pDC, Plasmacytoid Dendritic Cell; ROS, Reactive Oxygen Species; T Cell, T Lymphocyte; TGF-β, Transforming Growth Factor Beta; Th Cell, Helper T Lymphocyte; TLR, Toll Like Receptor; TNFα, Tumor Necrosis Factor Alpha; Treg, Regulatory T Lymphocyte.

Alongside the above-mentioned changes observed in the adipokine profile with the induction and progression of metabolic disease, a bi-directional interaction proceeds within the AT microenvironment among adipocytes and different types of resident and infiltrating immune cells. Details and outcomes of this interaction will be discussed for each of the cell types below. A summary of the different pathways and mediators involved is provided in Figure 1.

## Metabolic Regulation and Adaptation of Tissue Resident and Infiltrating Myeloid Cells

### Macrophages

Tissue-resident macrophages are highly heterogeneous with distinct, localization-dependent transcriptomes (135). Classically-activated M1 macrophages, which drive CLS formation, can be induced by LPS, toll-like receptor (TLR) ligands or interferon (IFN)-γ, secrete pro-inflammatory cytokines and upregulate the production of ROS and NO following activation (136). Conversely, alternatively-activated M2 macrophages, which contribute to AT homeostasis, are induced by IL-4 and IL-13, are implicated in the resolution of inflammation, and produce anti-inflammatory cytokines such as IL-10 (137). Although being useful to highlight the inflammatory state of tissues in health and disease, the M1/M2 macrophage classification paradigm is now considered an oversimplification as it does not consider microenvironmental factors.

Macrophages exhibit differential metabolic profiles based on their specific polarization and microenvironmental factors (138). These metabolic alterations are summarized in Table 1. Indeed, the dynamic inflammatory milieu of obese AT drives ATMs metabolic profile modifications. ATMs in obese mice exhibit an increased activation of OXPHOS and glycolysis (140). In addition, the activation of the NLRP3 inflammasome in macrophages by the increasing exogenous FAs in obesity contributes to the emergence of M1 macrophages (155–157). Moreover, monocytes and macrophages express the leptin receptor, which induces the proliferation of macrophages and the production of pro-inflammatory cytokines in response to leptin (158). In contrast to leptin, adiponectin suppresses the NF-κB-dependent expression of pro-inflammatory cytokines and promotes M2 polarization (53, 54). Nevertheless, another study argued that adiponectin induced the production of pro-inflammatory cytokines in M2 macrophages without interfering with their polarization (159).

**Table 1:** Metabolic pathways of classically activated M1 macrophages and alternatively activated M2 macrophages.

**Immune cell**	**Metabolic pathway**	**Metabolic reprogramming**	**Relevance of metabolic pathway to cellular function**	**Model**	**References**
M1 macrophages	Glycolysis	⇑	Inflammatory cytokines production	Myeloid-specific HIF-1α overexpressing mice	(139)
				HFD-fed mice and myeloid-specific HIF-1α^−/−^ mice	(140)
			Vascular remodeling	ATMs deletion in HFD-fed mice LPS and High Glucose stimulated macrophages	(141)
	Oxidative phosphorylation	⇓			(138)
	Pentose phosphate pathway	⇑	Production of inflammatory cytokines, ROS and NO	*Trypanosoma cruzi*-infected macrophages	(142)
			Inflammatory cytokines production	Myeloid-specific HIF-1α overexpressing mice	(139)
	TCA Cycle	Truncated	Production of prostaglandins, NO and ROS		(143)
	Lipogenesis	⇑	Required for Inflammasome activation and production of inflammatory mediators	Cecal ligation puncture-induced endotoxic shock in SREBP-1a-deficient mice LPS stimulated macrophages from mutant mice	(144)
			Required for Phagocytosis	SREBP-1a-deficient macrophages	(145)
			Required for inflammasome activation	Polymicrobial sepsis UCP2^−/−^ mouse model	(146)
	Glutamine metabolism	⇓	Required for polarization and T cell recruitment	Glutamine synthase (GS)-inhibited macrophages and GS^−/−^ macrophages	(147)
	Fatty acid oxidation	⇑	Inflammasome activation	NOX4-deficient mice	(148)
M2 macrophages	Glycolysis	⇑	Not essential for polarization unless OXPHOS is affected	BMDMs and Raw264.7 cells	(149)
	Oxidative phosphorylation	⇑	Required for polarization	BMDMs and Raw264.7 cells	(149)
	Glutamine metabolism	⇑	Required for polarization		(150)
	Fatty acid oxidation	⇑	Required for polarization and activation of the anti-inflammatory program	STAT6–/– BMDMs, embryonic-derived myeloid progenitors and PGC-1β transgenic mice	(151)
			Essentiality for polarization ?		(152–154)

⇑, *high metabolic rate*; ⇓, *low metabolic rate; ?, requires further investigation*.

### Dendritic Cells

Dendritic cells (DCs) are professional antigen-presenting cells that either instigate or suppress immune responses based on their maturation state. DCs are divided into two categories, plasmacytoid DC (pDC) and myeloid or conventional DC (cDC). Accumulating evidence implicates DCs and particularly cDCs in the regulation of AT inflammation. The DC population expands, promotes macrophage recruitment and induce a Th17-driven inflammatory response in HFD-fed mice (160–162). Indeed, HFD-fed mice exhibited an increased number of CD11c^+^ DCs in the AT whose ablation attenuated visceral AT inflammation (160, 161, 163). The accumulation of cDC during obesity was also attenuated in CCR7-deficient mice, which was associated with decreased AT inflammation (164, 165). Conversely, cDCs in AT were shown to acquire a tolerogenic phenotype through the activation of β-catenin and PPARγ without affecting weight gain (166). pDCs were also shown to accumulate in AT and to have detrimental effects in mice and humans (167, 168). DCs also influence the normal expansion of lean AT, where increased adiposity was accompanied by a reduction of CD11c^+^ AT DCs (169). Moreover, the uptake and accumulation of FAs in DCs and the formation of lipid droplets (LDs) were associated with increased DC immunogenicity (170, 171). Due to the lipid-rich environment of WAT especially in obesity, AT DCs are expected to acquire more LDs. Nevertheless, the functional impacts of this remain to be investigated. Different DC subsets exhibit distinct metabolic programs (172). In fact, resting and stimulated DCs have different metabolic requirements and thus, employ differential metabolic pathways. These pathways are highlighted in Table 2.

**Table 2:** Metabolic pathways utilized by activated dendritic cells.

**Immune cell**	**Metabolic pathway**	**Metabolic reprogramming**	**Relevance of metabolic pathway to cellular function**	**Model**	**References**
Activated DCs	Glycolysis	⇑	Required for activation	PAMPs-stimulated human monocyte-derived DCs	(173)
				TLR agonist-stimulated DCs	(174)
			Required for migration	*In vitro* stimulated bone marrow-derived DCs and splenic DCs	(175)
	Oxidative phosphorylation	-		PAMPs-stimulated human monocyte-derived DCs	(173)
		⇓			(176)
	Pentose phosphate pathway	⇑	Required for the enhanced synthesis of fatty acids		(177)
	TCA cycle	Truncated	Production of NO and ROS		(176)
	Lipogenesis	⇑	Required for activation	TLR agonist-stimulated DCs	(174)

⇑, *high metabolic rate*; ⇓, *low metabolic rate; -, no change in metabolic rate*.

### Neutrophils

Neutrophils are relatively rare in WAT of lean mice, where they are suggested to maintain tissue homeostasis (178). Neutrophils are among the first immune cells to be recruited to the AT of HFD-fed mice with a sustained infiltration. Neutrophils drive AT inflammation and IR through the production of inflammatory mediators and the formation of neutrophil extracellular traps (NETs) (179–181). Neutrophils accumulation in AT is dependent on the production of elastase, whose activity is enhanced in the AT of HFD-fed mice (179). WAT-infiltrating neutrophils exhibit an upregulation of IL-1β expression via NF-κB activation in an adipocyte contact-dependent manner (182). Adipocyte lipolysis and LTB4 production in WAT also accumulates neutrophils prior to macrophages and increases the production of IL-1β, which enhances macrophage recruitment into the AT (182). Nevertheless, it was proposed that neutrophils, similar to macrophages, exhibit phenotypic heterogeneity by which N1 neutrophils are pro-inflammatory and N2 neutrophils are anti-inflammatory (183, 184).

Neutrophils were believed not to require extensive metabolic networks and to solely depend on glycolysis as they exhibit a relatively low transcriptional and translational activity where (185). Nevertheless, novel evidence suggests the implication of the TCA cycle, OXPHOS, PPP, FAO, and glutaminolysis in neutrophil metabolism, demonstrating a broad metabolic plasticity (185) (Table 3).

**Table 3:** Metabolic pathways of neutrophils, eosinophils and mast cells.

**Immune cell**	**Metabolic pathway**	**Metabolic reprogramming**	**Relevance of metabolic pathway to cellular function**	**Model**	**References**
Neutrophils	Glycolysis	⇑	Required for phagocytosis	Stimulated human neutrophils *in vitro*	(186)
			Required for NETosis	Phorbol myristate acetate-stimulated human neutrophils *in vitro*	(187)
	Oxidative phosphorylation	⇑	Required for differentiation	shRNA induced knockdown of adenylate kinase 2 in neutrophil progenitor cells	(188)
			Required for chemotaxis and respiratory burst	Oligomycin and FCCP-treated human neutrophils	(189)
			Required for the production of ROS	LPS-treated mouse bone marrow-derived neutrophils treated with Antimycin A or myxothiazol	(190)
			Required for migration	Polg CRISPR/Cas9-mediated neutrophil-specific knockout in Zebra fish	(191)
	Pentose phosphate pathway	⇑	Required for NETosis	Amyloid fibril- and phorbol myristate acetate-stimulated human neutrophils	(192)
			Required for ROS generation and NETosis	G6PD-deficient patients G6PD-deficient mice	(193, 194)
	TCA cycle	⇑	Required for chemotaxis	Isocitrate dehydrogenase 1 mutant mice	(195)
			Required for differentiation	Mouse Atg5-deficient neutrophils and an *in vitro* model of differentiating neutrophils	(196)
	Lipogenesis	⇑	Required for differentiation	Atg7-deficient neutrophil precursors	(197)
			Required for neutrophil maintanence	FASlox/lox-Rosa26-CreER mice	(198)
	Glutamine metabolism	⇑	Not required for NETosis	Phorbol myristate acetate-stimulated human neutrophils *in vitro*	(187)
	Fatty acid oxidation	⇑	Required for NOX-2-dependent respiratory burst and ROS production	NOX deficient p47^−/−^ mice, bone marrow c-Kit^+/−^ neutrophils and human neutrophils	(199)
			Required for NETosis		(185)
			Required for differentiation	Atg7-deficient neutrophil precursors	(197)
Eosinophils	Glycolysis	⇑		Peripheral blood-derived human eosinophils	(200)
				IL-3, IL-5, or GM-CSF-stimulated human eosinophils	(201)
	Oxidative phosphorylation	⇑		Peripheral blood-derived human eosinophils	(200)
				IL-3, IL-5, or GM-CSF-stimulated human eosinophils	(201)
	TCA cycle	⇑		IL-3, IL-5, or GM-CSF-stimulated human eosinophils	(201)
	Glutamine metabolism	⇑		IL-3, IL-5, or GM-CSF-stimulated human eosinophils	(201)
Mast Cells	Glycolysis	⇑	Required for histamine release	2-DG-treated rat mast cells	(202)
			Required for IgE-mediated degranulation	High glucose-treated bone marrow-derived mouse mast cells	(203)
	Oxidative phosphorylation	⇑			(204)
	Pentose Phosphate Pathway	⇑			(204)
	TCA Cycle	Truncated	Accumulation of upstream intermediates that channel through the PPP Mast cell degranulation	Basophilic leukemia (RBL-2H3) cells and a mouse model of allergen-induced airway hyper-responsiveness	(204, 205)
	Lipogenesis	⇑			(204)

⇑, *high metabolic rate*; ⇓, *low metabolic rate*.

### Eosinophils

Eosinophils are multifunctional phagocytic granulocytes that are typically associated with helminth infection and allergic disorders (206). A growing body of evidence suggests a homeostatic role for AT-resident eosinophils (178). AT-resident eosinophils are sustained by AT multipotent stromal cells-derived CCL11 and ILC2-derived IL-5 (207, 208). Indeed, AT-resident eosinophils produce IL-4 and IL-13 that drive macrophage M2 polarization, trigger Th2 differentiation, enhance B cell activation and promote metabolic homeostasis (209, 210). Eosinophil-deficient HFD-fed mice showed pronounced IR (209, 211). Moreover, it was shown that HFD-induced adiposity can be inhibited by increasing the number of eosinophils in mice (207, 209). Conversely, another study demonstrated an increase in gonadal AT eosinophils in HFD-fed mice, which was supposed to be regulated by increased CCL11 expression (211). Indeed, HFD-fed Δdb1GATA and IL-5-KO mice lacking eosinophils or almost having no gonadal AT eosinophils, exhibited impaired insulin sensitivity (207, 212). The forced increase of AT eosinophils in different models demonstrated an enhanced metabolic homeostasis (209, 213, 214). Indeed, IL-4-stimulated eosinophils induced M2 macrophage polarization, while oxidized LDL-mediated induction promoted M1 macrophage polarization (209, 215, 216). HFD-fed transgenic mice overexpressing eotaxin2 specifically in AT exhibited an increased eosinophil migration into AT that was accompanied by enhanced glucose tolerance (217). It is also worth mentioning that leptin promotes while adiponectin attenuates eotaxin-induced human eosinophil adhesion and chemotaxis (55, 218, 219). Nevertheless, enhancing AT eosinophil abundance is debated since several studies demonstrated no beneficial or even negative outcomes of this approach (220).

It was suggested that circulating eosinophils display a greater metabolic flexibility in comparison to neutrophils (200, 201). Further investigation into the metabolic rewiring of eosinophils (shown in Table 3) is required as emerging roles of eosinophils suggest a central modulatory function in AT homeostasis.

### Mast Cells

Mast cells (MCs) are innate immune cells that originate from multipotent hematopoietic stem cells, then migrate to peripheral organs, where they undergo maturation giving rise to heterogeneous populations of mature MCs (21). MCs are enriched in visceral AT of mice and humans and are increased in settings of obesity and T2D, where they drive AT inflammation partly by enhancing macrophage infiltration (21, 221, 222). Indeed, the increased abundance of MCs in sub-cutaneous WAT of MetS subjects positively correlated with IR and markers of fibrosis and angiogenesis linking them to AT fibrosis and remodeling (223, 224). It was also suggested that MCs infiltration precedes the development of overt obesity (225). Indeed, the genetic ablation of MCs or manipulations impairing their function in HFD-fed mice resulted in decreased weight gain and reduced IR (221, 226, 227). Nevertheless, other studies utilizing different mouse models could not find a correlation between MC deficiency and the amelioration of AT inflammation (228, 229).

The activation of MCs is accompanied by a major metabolic reprogramming (Table 3). Indeed, these metabolic processes regulate MCs inflammatory cytokines and ROS production and IGE-mediated degranulation (203, 204, 230). A role for adipokines in the regulation of MC function has been recently revealed (56). Leptin and adiponectin were shown to exert opposite effects on MC polarization to promote a pro-inflammatory or an anti-inflammatory cytokine profile, respectively.

## Metabolic Regulation and Adaptation of Tissue Resident and Infiltrating Lymphoid Cells

### T Cells

T lymphocytes play major immunoregulatory and immunometabolic roles in AT homeostasis and dysfunction. Indeed, T cells were increased in VAT of obese mice and humans (231). Different effector T cells including helper T (Th) cells (T-bet-regulated Th1, GATA3-regulated Th2 and ROR-γt-regulated Th17) and cytotoxic T lymphocytes (CTLs) were shown to actively participate in obesity-associated WAT inflammation (178). Conversely, anti-inflammatory T cells such as regulatory T (Treg) cells and invariant natural killer T (iNKT) cells that reside in the AT under physiological conditions were reduced in obesity (232, 233). Based on the composition of the T-cell antigen receptors (TCR), T cells are categorized into two populations, αβ T and γδ T cells. αβ T cells are further classified, based on their surface markers, into CD4^+^ T cells and CD8^+^ T cells that upon activation, differentiate into Th cells and CTLs, respectively. Tregs emerge as a subset of CD4^+^ T cells that negatively regulate immune responses with a characteristic signature CD4^+^ CD25^+^ Foxp3^+^.

#### αβT Cells

αβ T cells represent the second largest immune population in WAT (178). In obesity, T cells are enriched in visceral AT of mice and humans, and are possibly recruited through a CCR5-CCL5-mediated interaction (231, 234). It was proposed that T cells infiltration precedes that of macrophages (235). However, this is debated as other studies did not arrive at a similar result (236, 237). CD4^+^ T cells represent the more abundant subtype in visceral AT and are further enriched in obesity (234, 238). In addition to their recruitment from the general circulation, both CD4^+^ and CD8^+^ T cells undergo clonal expansion in epicardial WAT (239, 240). MHCII expression was increased post-HFD feeding and mice deficient in MHCII exhibited greater insulin sensitivity (241). Nevertheless, inhibiting MHCII in HFD-fed mice did not improve glucose tolerance, an improvement seen with conventional T cells deficiency (242–244). The depletion of CD8^+^ cells in HFD-fed mice decreased the expression of TNF-α and IL-6 in epicardial WAT, which was accompanied by an enhanced glucose and insulin tolerance (235). Similarly, CD4^+^ Th1 cells were shown to drive AT inflammation and glucose intolerance (245). In fact, Th1 cells are similar in proportion to Tregs in lean conditions, while they occur at a higher frequency in comparison to other CD4^+^ cell subtypes in obesity (242, 246). Similarly, Th17 cells accumulated in sub-cutaneous WAT of insulin resistant individuals (247). IL-17-deficient mice displayed a better insulin and glucose tolerance, this was however abrogated by HFD feeding (248). Th2 cells were shown to have a beneficial effect on AT inflammation. Rag-deficient HFD-fed mice showed marked obesity and IR in comparison to their WT counterparts, a phenotype that was abrogated by the adoptive transfer of CD4^+^, but not CD8^+^ T cells (242). Indeed, most of CD4^+^ cells that homed to epicardial WAT expressed GATA3 (242).

Increasing evidence suggests a pivotal role for metabolic pathways in naïve and activated T cells maintenance and function (249). On activation, naïve T cells undergo major metabolic reprogramming, which highly depends on the duration and strength of TCR stimulation, including glucose metabolism, glutamine metabolism, and biosynthetic pathways (249). These metabolic alterations are summarized in Table 4.

**Table 4:** Metabolic pathways required for T lymphocyte proliferation, differentiation, function, and activity.

**Immune cell**	**Metabolic pathway**	**Metabolic reprogramming**	**Relevance of metabolic pathway to cellular function**	**Model**	**References**
αβ T cells	Glycolysis	⇑	Required for cell growth and clonal proliferation		(249)
				Glut1 transgenic mice Murine model of asthma	(250)
	Oxidative phosphorylation	⇑	Required for the survival, proliferation, generation and function		(249)
	Pentose phosphate pathway	⇑	Nucleotide and ribosome biosynthesis		(249)
	TCA Cycle	⇑			(249)
	Lipogenesis	⇑	Required for Th17 development, production of membrane phospholipids and inflammatory function	Pharmacological and genetic inhibition of ACC1 in mice Human T cell cultures Murine model of experimental autoimmune encephalomyelitis	(251)
			Required for growth and proliferation		(249)
	Glutamine Metabolism	⇑	Regulates T cell activation		(249)
	Fatty Acid Oxidation	⇑		*Ex vivo* human CD4^+^CD25^−^Foxp3^−^	(252)
		⇓		Glut1 transgenic mice Murine model of asthma	(250)
Regulatory T Cells	Glycolysis	⇑	Required for cellular migration	Treg-specific HIF-1α^−/−^ mice	(253)
			Required for cellular migration but not immunosuppressive function	Foxp3-GFP and Cd28^Y170F^ genetically targeted mice on C57BL/6 background Ctla4^−/−^ mice Murine lung microvascular endothelial cells, bone marrow-derived dendritic cells H2-d allospecific Treg cells Loss-of-function GCK mutation human blood samples	(254)
			Required for proliferation	*Ex vivo* CD4^+^CD25^hi^Foxp3^+^CD127^−^ Treg cells	(252)
	Lipogenesis	⇓		Pharmacological and genetic inhibition of ACC1 in mice Human T cell cultures Murine model of experimental autoimmune encephalomyelitis	(251)
	Fatty Acid Oxidation	⇑	Required for proliferation	*Ex vivo* CD4^+^CD25^hi^Foxp3^+^CD127^−^ Treg cells	(252)
			Required for immunosuppressive activity	Treg-specific HIF-1α^−/−^ mice	(253)
				Glut1 transgenic mice Murine model of asthma	(250)
γδ T cells	Glycolysis	⇑	In γδ T1 cells and is required for differentiation and cytokine production	CD2-cre;Raptor-f/f mice	(255)
	Oxidative phosphorylation	⇑	In γδ T17 cells and is required for the production of IL-17	MyD88^−/−^, Il1r1^−/−^ and IL-23R KO and conditional (CD2-cre; Raptor^fl/fl^, CD-2-cre;Rictor^fl/fl^ and CD-2-cre;Stat3^fl/fl^) KO miceHuman subjects with psoriasis vulgarisPsoriasis-like mouse model	(256)
	TCA cycle	⇑	Required for the production of IL-17	MyD88^−/−^, Il1r1^−/−^, and IL-23R KO and conditional (CD2-cre; Raptor^fl/fl^, CD-2-cre;Rictor^fl/fl^ and CD-2-cre;Stat3^fl/fl^) KO mice Human subjects with psoriasis vulgaris Psoriasis-like mouse model	(256)
iNKT cells	Glycolysis	⇑	Required for the production of IFN-γ and TCR recycling and accumulation in the immune synapse	Murine spleen and liver Vα14 Tg.cxcr6^gfp/+^ iNKT cells	(257)
	Oxidative PHOSPHORYLATION	⇓		Murine spleen and liver Vα14 Tg.cxcr6^gfp/+^ iNKT cells	(257)
		⇑	Essential for survival, proliferation and selective cytokine production	PLZF^+/−^ and PLFZ^Tg^ mice spleen NKT cells	(258)
	Pentose phosphate pathway	⇑	Required for effector functions	PLZF^+/−^ and PLFZ^Tg^ mice spleen NKT cells	(258)
	Lipogenesis	⇑	Required for the production of IFN-γ	Murine spleen Vα14 Tg.cxcr6^gfp/+^ iNKT cells Clinical tumor biospecimens from HCC patients	(259)

⇑, *high metabolic rate*; ⇓, *low metabolic rate*.

#### Regulatory T Cells

Phenotypically-distinct AT-resident Tregs were reported to be enriched in visceral AT of lean mice, where they originate from enhanced proliferation rather than circulating Tregs infiltration (178). Visceral AT Tregs are markedly reduced in obese mice and humans, which promotes inflammation (232, 260, 261). Conversely, expanding Tregs in HFD-fed mice improved metabolic parameters (262). Indeed, PPARγ is essential for the accumulation and function of Tregs in AT of lean mice, where it collaborates with Foxp3 to induce their distinct phenotype, a phenotype abrogated by obesity through phosphorylating PPARγ at position Ser273 (263–265). Moreover, it was shown that IL-33/ST-2 axis amplified Tregs in visceral AT, which was accompanied by an attenuation of inflammation in obese mice (266, 267). Indeed, Tregs are highly enriched in visceral AT and to a lesser extent in sub-cutaneous WAT, where IL-33 is expressed (268–270). Other immune cells including γδ T cells, ILC2s and iNKT cells were also shown to regulate AT Tregs accumulation promoting insulin sensitivity (178). Conversely, IFN-γ producing Th1 cells inhibited AT Tregs and thus promoted IR (271).

The development, function, and phenotype stabilization of Tregs is metabolically regulated by several pathways highlighted in Table 4 (272). Moreover, leptin metabolism was shown to partially induce Tregs *in vitro* and mice deficient in leptin exhibited an increased proliferative ability of Tregs (273). Several lines of evidence suggest that Treg deficiency or insufficiency can lead to both T1D and T2D (274). Although studies demonstrated no difference in Treg frequency in diabetes, Treg phenotype and suppressive function were altered (275).

#### γδT Cells

γδ T cells can be classified into two major functional groups, IFN-γ-producing γδ T1 cells and IL-17-producing γδ T17 cells (178). In comparison to αβ T cells, γδ T cells harbor a restricted TCR repertoire, and the antigens recognized by these cells remain largely unknown. γδ T cells are as abundant as MCs, neutrophils, and CD8^+^ T cells in lean WAT, and are increased in response to HFD consumption (268, 276). Earlier investigations into the role of γδ T cells in AT demonstrated a pro-inflammatory function in HFD-fed mice (276). Nevertheless, circulating γδ T cells were decreased in obese subjects and were negatively correlated with BMI (277). More recently, γδ T cells of epicardial WAT were shown to comprise two distinct populations; PLZF^−^, CD3ε^low^, CD27^+^, RORγT^−^, T-bet^+^ γδ T cells that produce IFN-γ and PLZF^+^, CD3ε^high^, CD27^−^, RORγT^+^, T-bet^−^ cells that produce TNF-α and IL-17A (268). Indeed, mice deficient in PLZF^+^ γδ T cells or IL-17A KO mice exhibited reduced IL-33 levels and failed to accumulate ILC2s and Tregs in AT, suggesting that PLZF^+^ γδ T cell-produced cytokines modulate the number of IL-33-producing stromal cells. Moreover, PPARβ overexpressing mice, which exhibited lower αβ T cells and higher γδ T cells, were protected from HFD-induced AT inflammation and IR (278). Intriguingly, it was demonstrated that γδ T cells were initially increased in AT of ketogenic diet-fed mice but then decreased following the development of obesity (279). Table 4 highlights the fact that different γδ T cell subtypes exhibit a differential metabolic profile depending on their polarization (256).

#### iNKT Cells

iNKT cells represent a subset of the innate-like T lymphocytes, NKT cells, that recognize glycolipids presented on MHC-I-like family protein CD1d and express a conserved semi-invariant TCR that recognizes the prototypic ligand α-galactosylceramide (178). Indeed, adipose iNKT cells exhibit a distinct transcriptome from that of iNKT cells residing in other tissues with both anti-inflammatory and pro-inflammatory characteristics, secreting Th1-recruiting IFN-γ and Th2-recruiting IL-4 (280, 281). iNKT cells are suggested to modulate WAT immunity in setting of leanness and obesity (282, 283). iNKT cells were enriched in visceral AT of humans and mice and in mouse sub-cutaneous WAT, where CD1d-expressing M2 macrophages and adipocytes promptly activate iNKT cells (283–285). WAT iNKT cells contribute to metabolic homeostasis through the secretion of IL-2 and IL-10, which regulate M2 macrophages and Tregs function, respectively (283). In obesity, the number of AT iNKT cells decline with WAT inflammation (284–288). Also, iNKT cells were shown to be dysfunctional in patients suffering from obesity or T2D exhibited by a diminished capacity to secrete IL-2 (289). Alternatively, HFD-fed mice deficient in AT iNKT cells were prone to obesity and IR which were reversed upon the adoptive transfer of iNKT cells (290, 291). Importantly, the hypoxic condition of expanding AT favors the upregulation of HIF-1α (10, 292). iNKT cells respond to hypoxia by upregulating the CD1d-mediated cytokine response (293). Furthermore, leptin activates iNKT cells resulting in their anergy and PD-1 upregulation (294, 295). Moreover, the inhibition of the synthesis of glucosylceramide, which can be presented on CD1d, in adipocytes was shown to impair iNKT cell activity and cytokine production (296). Several lines of evidence suggest that iNKT cell metabolism contributes to their development and functioning (shown in Table 4).

#### Innate Lymphoid Cells

Innate lymphoid cells have been previously regarded as enigmatic lymphocyte-like cells that possess the morphological features of a lymphocyte in the immature state but lack its surface markers, and are thus described as “lineage negative.” ILCs include three transcriptionally-defined groups; Tbet-dependent ILC1s (which include NK cells) that secrete IFN-γ and TNF-α, GATA3-dependent ILC2s that secrete IL-5/IL-13 and IL-10, ROR-γt-dependent ILC3s that secrete IL-17A/IL-22 and finally Id3-dependent ILCregs that produce IL-10 and require autocrine TGF-β1 (297). Importantly, recent evidence demonstrated the presence of all ILC subsets in different AT depots, where they are implicated in AT immune responses (298).

##### Adipose Tissue ILC1s and NK Cells

AT-resident ILC1s and NK cells are highly enriched in WAT in both humans and mice, and further increase at the setting of obesity and T2D, where they positively correlated with IR (299, 300). ILC1s and NK cells drive AT inflammation in obesity by secreting IFN-γ and promoting M1 macrophage polarization (299, 301). However, the enrichment of ILC1s and NK cells in WAT at steady state suggests homeostatic roles (302). Indeed, ILC1s and NK cells were shown to regulate the survival of ATMs by killing AT M2 macrophages (302). Nevertheless, the physiological relevance of this regulation is questioned since mice and human deficient in ILC1s do not display major metabolic derangements (303, 304). ILC1s and NK cells exhibit a distinct metabolic program following activation. These alterations drive cellular functions, cytotoxicity, and inflammatory cytokines production (305–307). Table 5 highlights metabolic pathways implicated in ILC1s and NK cells activity and function.

**Table 5:** Metabolic pathways of innate lymphoid cells.

**Immune cell**	**Metabolic pathway**	**Metabolic reprogramming**	**Relevance of metabolic pathway to cellular function**	**Model**	**References**
ILC1s and NK Cells	Glycolysis	⇑	Required for cytotoxicity and IFN-γ production Not required for NK cell degranulation	IL-2 or IL-12/15-stimulated peripheral blood NK cells	(308)
			NK cell proliferation and cytotoxicity	IL-15-activated NK cells *in vitro* and MCMV infection in mice	(309)
	Oxidative Phosphorylation	⇑		IL-2 or IL-12/15-stimulated peripheral blood NK cells	(308)
				Primary murine NK cells	(310)
			Not required for IFN-γ production	IL-12 and IL-18-stimulated primary murine NK cells	
			Required for NK cell activation	NK receptor-activating stimulation of primary murine NK cells	
ILC2s	Glycolysis	⇓	Required for proliferation and cytokines production	Arg1-deficient ILCs in mice	(311, 312)
			?	Arg1-deficient ILC2s in a mouse model of helminth infection	(56)
			Required for ILC2 development	Conditional deletion of E3 ubiquitin ligase VHL in innate lymphoid progenitors	(313)
			Required for ILC2 homeostasis and cytokine production	Atg5^−/−^ mice	(314)
	Fatty Acid Oxidation	⇑	Required for accumulation and production of IL-13 and IL-5	Rag1^−/−^ mouse model of helminth infection and malnutrition	(315)
			Required for ILC2 homeostasis and cytokine production	Atg5^−/−^ mice	(314)

⇑, *high metabolic rate*; ⇓, *low metabolic rate; ?, require further investigation*.

##### Adipose Tissue ILC2s

ILC2 are key regulators of lean AT homeostasis (298). ILC2s are enriched in visceral AT, where they represent the predominant producers of IL-5 and IL-13 which are essential for the recruitment of eosinophils (207). Indeed, the recruitment and proliferation of AT ILC2s is driven by IL-33 whose origin is still debated (297, 316). Moreover, AT ILC2s express ICOSL, which signals to Tregs through ICOS and drive their accumulation in visceral AT at steady state, a process abrogated in obesity by IFN-γ (317). Moreover, ILC2s upregulate OX40L following their stimulation by IL-33 which is essential for the recruitment of Treg cells into the AT (318). The exact mechanisms leading to the reduction of AT ILC2s number in obesity is not well-understood. Nevertheless, one possible mechanism includes the expansion of ILC1s where ILC1-derived IFN-γ antagonizes ILC2s (317). Another mechanism implicates IL-12 in driving the conversion of ILC2s to ILC1s in the context of diet-induced obesity (DIO) (319). Metabolic pathways in ILC2s govern their proliferation and function. Shifting the balance between OXPHOS and glycolysis toward glycolysis impairs the development and function of ILC2s (311, 312). Metabolic pathways implicated in ILC2s metabolism are summarized in Table 5.

### B Cells

B lymphocytes are further subdivided into 2 major classes; B-1 and B-2 depending on their developmental origin, microenvironmental niches and the requirement of Th cells to produce antibodies (320). B-1 cells are further stratified to B1-a and B1-b cells, which differ by their surface expression of CD5. B cell-secreted IL-10, IL-35 and TGF-β are characteristics of the functionally-distinct Breg cells that can derive from both B-1 and B-2 cells. Breg cells suppress Th1 and Th2 polarization, and inhibit macrophage and dendritic cell activation and cytokine production. B-1 and B-2 cells coexist in perivascular AT, epicardial WAT and BAT, where the B1:B2 ratio is higher than that in sub-cutaneous WAT but yet varies greatly in a depot-specific manner (321, 322). B cells are among the first immune cells to infiltrate AT following the consumption of a HFD consistent with an increased IR, where AT B-2 cells are thought to promote inflammation (323–327). B cell abundance also increases in BAT following the consumption of a HFD, where their role is poorly understood (328). Indeed, B cells global deficiency in mice attenuated HFD-induced AT inflammation and reduced IR (324, 325). Consistently, circulating B cells from obese, diabetic and obese diabetic individuals produced higher amounts of pro-inflammatory cytokines in comparison to healthy individuals (329, 330). In addition, B cell-produced pro-inflammatory immunoglobulins were elevated in visceral AT of obese mice activating macrophages to secrete inflammatory cytokines (324, 331). Contrary to B-2 cells, B-1 cell-derived natural IgG and anti-inflammatory cytokines block AT inflammation and improve glucose tolerance through inducing M2 macrophage polarization and increasing their production of IL-10, while reducing their production of IL-6 and TNF-α (321, 322, 332–335). The number of Breg cells, which are present in AT, is reduced in diabetic patients in comparison to healthy donors (336, 337). Moreover, Breg cells from diabetic patients secrete less IL-10 (329, 330). Indeed, the adoptive transfer of Breg cells ameliorated AT inflammation and IR in DIO mice (336). Different B cell subsets exhibit distinctive metabolic profiles depending on their particular microenvironments and thus, careful interpretation of B cell metabolic data is pivotal (338). The metabolic pathways implicated in B cell metabolism are summarized in Table 6. Metabolic rewiring of different subsets of B cells and plasma cells have also been discussed in details elsewhere (338).

**Table 6:** Metabolic pathways implicated in B lymphocyte proliferation, differentiation, activation, and function.

**Immune cell**	**Metabolic pathway**	**Metabolic reprogramming**	**Relevance of metabolic pathway to cellular function**	**Model**	**References**
B cells	Glycolysis	⇑	Required for proliferation and antibody secretion	Rag1^−/−^ mice LPS, anti-IgM or CpG oligodeoxynucleotide-stimulated mouse B cells B cell-specific Glut1 deletion Chronic BAFF stimulation of B cells	(339)
			B cell antigen receptor (BCR)-mediated growth	p85α-deficient mice	(340)
			Limited time frame BCR-mediated metabolic activation	Anti-IgM and CpG-stimulated mouse B cells B cells of MD4 mice treated with oligomycin, 2-DG and FCCP	(341)
		⇓	In germinal center B cells	*Ex vivo* germinal Center B cells Cpt2-knockdown B cells	(342)
	Oxidative phosphorylation	⇑	Required for B cell growth and differentiation	IL-4-stimulated and oligomycin-treated primary mouse B cells	(343)
			Limited time frame BCR-mediated metabolic activation	Anti-IgM and CpG-stimulated mouse B cells B cells of MD4 mice treated with oligomycin, 2-DG and FCCP	(341)
	Pentose phosphate pathway	⇑		p85α-deficient mice	(340)
	tca cycle	⇑		IL-4-stimulated and oligomycin-treated primary mouse B cells	(343)
		⇓	In Germinal Center B cells	*Ex vivo* germinal Center B cells Cpt2-knockdown B cells	(342)
	Lipogenesis	⇑	Proliferation and expansion of endomembrane network in response to LPS	LPS-stimulated murine splenic B lymphocytes CH12 B lymphoma cells	(344)
	Glutamine metabolism	⇑	Required for B cell growth and differentiation	IL-4-stimulated and oligomycin-treated primary mouse B cells	(343)

⇑, *high metabolic rate*; ⇓, *low metabolic rate*.

## Immune Cell Contribution to Depot-Specific Adipose Tissue Inflammation

While AT is broadly classified in WAT and BAT, WAT is further divided into several distinct depots that differ in their properties and microenvironments including subcutaneous (scWAT) and visceral WAT (VAT) depots. The latter includes epicardial (EpiCAT), perivascular (PVAT), epidydimal (EpiWAT), mesenteric (MAT) and perirenal (PRAT) AT. VAT has been extensively studied due to the association between visceral obesity and the emergence of CVD risks (345, 346). It was demonstrated that scWAT exhibits a greater potential than VAT to undergo beiging, a process by which white adipocytes become brown-like and participate in energy dissipation (2, 347). Indeed, the induction of VAT beiging has been largely regarded as an approach to curb obesity and its accompanying metabolic and cardiovascular derangements (348, 349). Nevertheless, several visceral adipose depots including PVAT and EpiCAT intrinsically possess a beige phenotype and the implications of thermogenic induction in these particular tissues on cardiovascular functioning is not yet well-characterized, especially that the immune landscape of these tissues is less known (33). In the below sections, an overview of changes in immune cell function, population, and activity, as well as the alterations in adipokine and cytokine profile across different depots will be provided with particular emphasis on PVAT and EpiCAT due to their relevance to CVD in metabolic impairment.

### Subcutaneous Adipose Tissue

The mass of scWAT is positively correlated with BMI in obese subjects (350). A dysregulated scWAT in patients with MetS exhibits higher macrophage infiltration and CLS formation, which is accompanied by a dysregulated adipokine profile (351). Indeed, scWAT inflammation is linked to the development of IR (214, 352). Interestingly, a sustained scWAT low-grade inflammation extending beyond weight loss was reported (353, 354). This sustained inflammation has been attributed to the accumulation of effector memory T cells (354). This contradicts with recent reports that demonstrated VAT but not scWAT inflammation as a manifestation of obesity (355, 356). Such discrepancies may arise from differences in diet composition and the duration of HFD-feeding. The general consensus however is that scWAT inflammation participates in driving IR and the MetS in obese subjects with the implication of various immune cells.

It was shown that an increased abundance of eosinophils in scWAT of MetS patients, is accompanied with IR, tissue fibrosis, and adipokine dysregulation (357). Macrophage infiltration and CLS formation are also elevated in scWAT of obese and diabetic patients (351, 358). Interestingly, an accumulation of M2 macrophages in scWAT but not in VAT of obese patients is associated with inflammation limitation (359). Moreover, the abundance of MCs is increased in scWAT of MetS subjects and is significantly correlated with IR, leptin, IL-1β, IL-6, and the activities of MAPK and NF-κB in circulating monocytes (224). Similar to eosinophils, scWAT MCs are correlated with markers of AT fibrosis and angiogenesis (224). The number of total dendritic cells is reduced but that of pDC increases in the scWAT of subjects with T2D implicating pDCs in scWAT low-grade inflammation (360).

### Epidydimal Adipose Tissue

EpiWAT is a metabolically active visceral fat pad, which is anatomically attached to the testis and epididymis, then stretches out toward the diaphragm (361). EpiWAT low-grade inflammation is thought to contribute to the initiation of IR, MetS and its related cardiovascular derangements (362). Indeed, macrophage infiltration and accumulation in EpiWAT is at the core of this inflammation (222, 362). Interestingly, Chronic DIO eventually leads to decreased EpiWAT mass, which correlated negatively with body weight and was associated with a widespread of CLSs and MCs, together with an impaired adipokines gene expression, which could be attributed to the increased abundance of dysfunctional or dead fat cells (361).

EpiWAT neutrophils of HFD-fed mice exhibited an increased IL-6 expression in EpiWAT due to an adipocyte-contact-dependent activation of NF-κB (182). Moreover, it was suggested that HFD-induced EpiWAT fibrosis was attributed to macrophages and MCs (222). MCs were also shown to have a role in HFD-stimulated adipocytes senescence in EpiWAT (222). It has also been shown that the consumption of a HFD induces an elevation of NK count and the production of pro-inflammatory cytokines in EpiWAT at an early phase of obesity induction, which was linked to increased fasting glucose, insulin levels and ATMs count (301). Interestingly, NK-mediated EpiWAT derangements were IFN-γ-dependent at early stages and then became TNFα-dependent (363). Moreover, studies revealed an increase in B cells in EpiWAT of HFD-fed mice, which was associated with IR (320). These B cells were shown to drive VAT inflammation by regulating the activity of VAT T cells and macrophages through the production of pro-inflammatory cytokines including IL-6 and INF-γ (320). HFD induces an increase of the immature DCs count in EpiWAT, which has a role in adipose tissue inflammation through triggering a parallel increased production of Th17 cells via promoting an excessive production of IL-6, TGF-β, and IL-23 (162).

### Perivascular Adipose Tissue

PVAT environs most of the blood vessels including the aorta and coronary and subcutaneous small arteries. PVAT play a crucial role in supporting blood vessels by maintaining vasomotor tone and insulating them from surrounding environment (364). It communicates with neighboring VSMCs and ECs in a paracrine manner through the production of various adipokines influencing the vascular tone (365). Indeed, PVAT-derived adipokines infiltrate into vasculature and serve as either vasodilators or vasoconstrictors. As such, an adipokine profile imbalance in PVAT toward proinflammatory adipokines is suggested to drive vascular derangements in metabolic disorders through the disruption of PVAT anticontractile activity. Furthermore, PVAT inflammation has been reported in states of nutritional excess through the recruitment of pro-inflammatory cells. This interaction provides the framework by which PVAT inflammation impairs vascular function (366–369).

PVAT infiltrating immune cells include macrophages, T cells, NK cells, and DCs that produce both inflammatory and anti-inflammatory cytokines, depending on the adipokine profile shifts (363, 370–373). Interestingly, both B cell subtypes occur in PVAT, where B-2s promote the development of diet-induced atherosclerosis and B-1s inhibit it by reducing MCP-1 and TNF-α production (334, 374, 375). However, PVAT possesses a higher B1:B2 ratio and thus, B cells have an anti-inflammatory role in PVAT (322). Recent studies done on rat PVAT inflammation demonstrated that the increased production of IL-1β and TGF-β1 was correlated with a reduced AMPK activity and endothelial relaxation impairment (32). On the other hand, there was an increase in AT hypertrophy, oxidative stress, and Rho-associated kinase (ROCK)-mediated Ca^2+^ sensitivity (32, 376). Other factors associated with PVAT inflammation include adipocyte derived MCP-1 and low-density lipoprotein receptor related protein-1 (377, 378).

On the other hand, several PVAT adipokines were reported to have an ameliorative effect on vascular function. Indeed, the treatment with the adipokine irisin normalizes the reduced anti-contractile properties of aortic PVAT in obese mice (379). Adiponectin was also shown to attenuate vascular inflammation and atherosclerosis possibly through blocking NF-κB signaling and downregulating the expression of VCAM-1 and ICAM-1 (380–383). Moreover, PVAT-derived adiponectin normalizes endothelial function partly through enhancing endothelial eNOS phosphorylation (384). Similarly, omentin was suggested to have an anti-atherogenic potential in concert with circulating adiponectin (385). In fact, omentin was demonstrated to restore endothelium-dependent relaxation by inhibiting ROS and enhancing NO production in high glucose-treated endothelial cells (386, 387).

**Figure 2:**
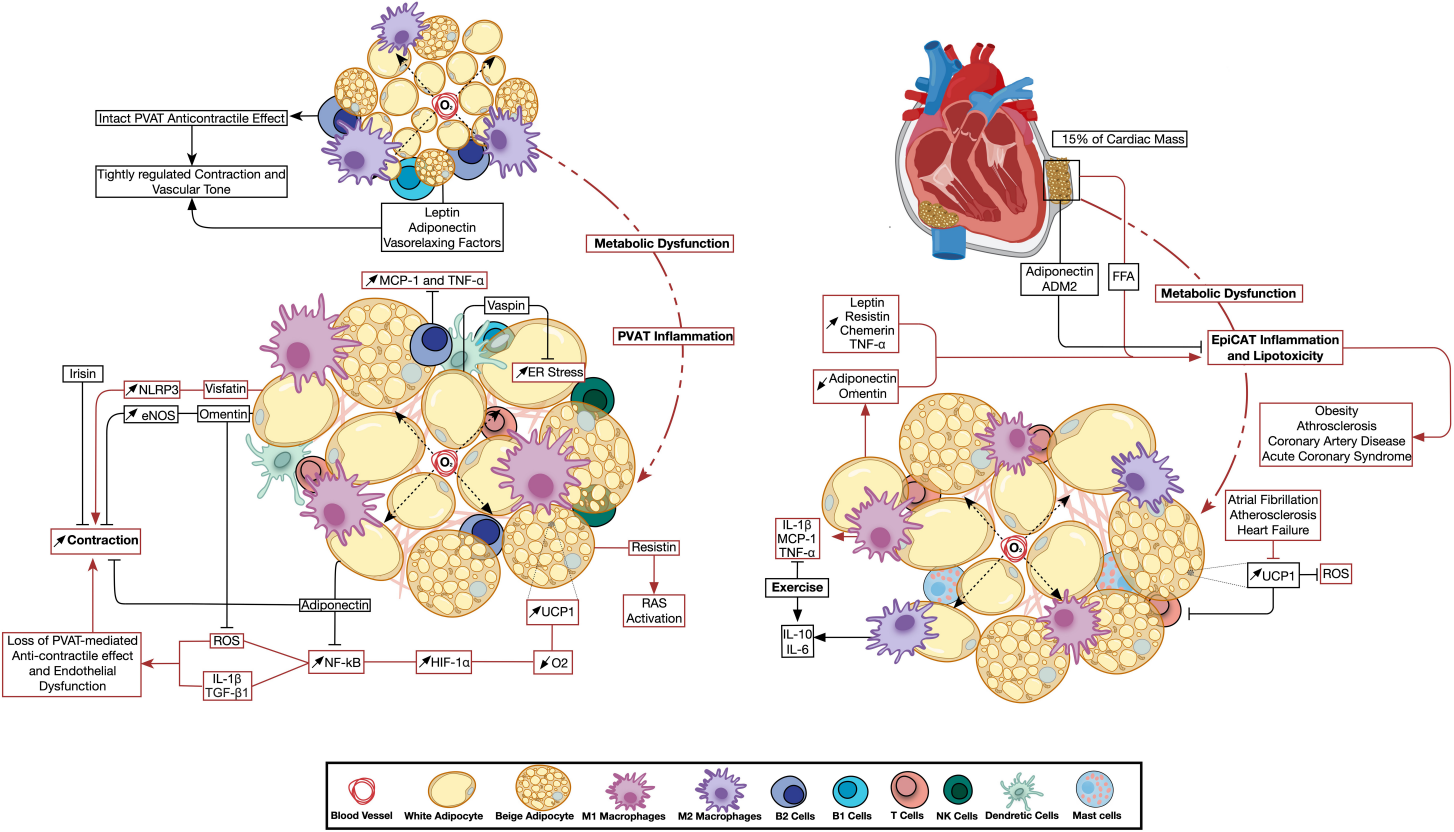
Perivascular and epicardiac adipose tissue dysfunction: the emerging role of immune cell and adipokine profile dysregulation. Metabolic impairment triggers changes in PVAT (left) and EpiCAT (right) adipokine environment and immune cell activity. Inflammation has a direct effect on the neighboring vascular and cardiac tissue. Changes in UCP1 expression were reported to have opposite effects in either depot. Pathways active in basal PVAT and EpiCAT homeostasis are depicted in black arrows, while those activated during inflammation are shown in red. ADM2, Adrenomedullin-2; eNOS, Endothelial Nitric Oxide Synthase; EpiCAT, Epicardial Adipose Tissue; ER Stress, Endoplasmic Reticulum Stress; HIF-1α, Hypoxia-induced Factor 1 Alpha; IL, Interleukin; MCP-1, Monocyte Chemoattractant Protein 1; NF-κB, Nuclear Factor Kapp-light-chain-enhancer of Activated B cells; NLRP3, NLR Family Pyrin Domain Containing 3; O2, Oxygen; PVAT, Perivascular Adipose Tissue; RAS, Renin Angiotensin System; ROS, Reactive Oxygen Species; TGF-β, Transforming Growth Factor Beta; TNFα, Tumor Necrosis Factor Alpha; UCP1, Uncoupling Protein 1.

Other PVAT-derived adipokines include vaspin, which modulates ER stress by upregulating the phosphorylation of Akt and AMPK (388), and apelin, which maintains vascular structure by upregulating endothelial NO (389, 390). Despite the positive correlation between serum leptin levels and vascular calcification (391), PVAT-derived leptin was reported to exert an anticontractile effect when in synergy with other vasorelaxing factors (392, 393). Nevertheless, PVAT-derived leptin also promotes VSMC phenotypic switch by increasing the phosphorylation of p38 MAPK (394–396). Chemerin was demonstrated to promote aortic atherosclerosis by promoting NF-κB signaling and p38 MAPK phosphorylation (397, 398). Additionally, and via the activation of NLRP3 signaling, the adipokine visfatin was shown to induce vascular endothelial dysfunction and tissue inflammation (399, 400). Finally, resistin was demonstrated to activate the renin-angiotensin system inducing hypertension (401, 402). Changes in PVAT adipokine environment and immune cell activity brought about by metabolic dysfunction is depicted in Figure 2.

### Epicardial Adipose Tissue

EpiCAT is located in the atrioventricular and interventricular heart grooves and plays a role in providing FA to the myocardium. Indeed, EpiCAT represents 15% of the cardiac mass, and as the epicardial fat increases, the ventricles and the epicardial surfaces get covered by EpiCAT. Moreover, EpiCAT surrounds the adventitia of coronary arteries and plays a cardioprotective role during metabolic and mechanical insults (33). The endocrine function of EpiCAT has witnessed extensive investigation as EpiCAT dysfunction was implicated in cardiovascular diseases. It regulates FA homeostasis to prevent lipotoxicity, while secreting anti-inflammatory and anti-atherogenic adipokines under healthy conditions (403, 404). However, EpiCAT alters its adipokines to release FA and pro-inflammatory cytokines under metabolic insults (404, 405). Several studies reported the expression of numerous adipokines in EpiCAT including adiponectin, omentin, adipsin, leptin, resistin, visfatin, chemerin and adrenomedullin (406). While the EpiCAT expression of resistin, leptin and TNF-α increase in obesity, the expression of adiponectin is markedly reduced (407, 408). In addition, EpiCAT adiponectin expression is also decreased while that of leptin increased in CAD patients (409, 410). Importantly, the administration of recombinant adiponectin can reverse the harmful effects of dysfunctional EpiCAT-derived factors (409). Another study demonstrated a differential expression of adiponectin, visfatin, leptin, chemerin and vaspin in periaortic, pericoronary and apical EpiCAT, where these adipokines were correlated with either aortic or coronary atherosclerosis (411). Furthermore, the expression level of omentin was decreased in CAD patients (412). Importantly, it was suggested that exogenous omentin supplementation might support a cardioprotective role through its anti-inflammatory effect on EpiCAT (413). Increased levels of resistin in EpiCAT were also reported in patients with advanced coronary atherosclerosis and patients with acute coronary syndrome (414, 415). Similarly, higher chemerin levels were observed in CAD patients which was correlated with an increased EpiCAT volume (416, 417). Interestingly, the level of chemerin was positively correlated with the severity of coronary atherosclerosis in CAD patients (416).

Immunohistochemistry done on EpiCAT confirmed the presence of infiltrating CD3^+^ T cells, tryptase^+^ mast cells, and CD68^+^ macrophages. These immune cells have been shown to be unique to EpiCAT when compared to scWAT (418). A study showed that patients with coronary artery disease (CAD) had a significant increase in macrophage infiltration into EpiCAT compared to individuals without CAD (419). Furthermore, the levels of IL-6, IL-1β, MCP-1, and TNF-α were higher in EpiCAT compared to scWAT (418). Changes in EpiCAT adipokine environment and immune cell activity brought about by metabolic dysfunction is depicted in Figure 2.

### Mesenteric Adipose Tissue

MAT is located between the gut and the liver. Several lines of evidence associate MAT expansion to an elevated risk for the development of peripheral and central IR as well as CVDs (327, 420). In response to HFD consumption, MAT adipocytes secrete high amounts of MCP-1, which intensifies the inflammatory response by modulating macrophage infiltration driving IR and atherosclerosis (421). Similar to MCP-1, GM-CSF is highly expressed in MAT of obese animals. It has been shown that both GM-CSF and B cells play an important role in the activation and accumulation of macrophages besides the production of pro-inflammatory cytokines in MAT of HFD-fed animals (327, 422). It is worth noting that B cells are among the earliest immune cells infiltrating MAT in HFD-induced AT inflammation models (327). Likewise, an increased accumulation of mast cells was shown in MAT of HFD-fed mice, which was associated with tissue fibrosis and IR. These alterations occurred coincidently with the progression of obesity and diabetes (225).

### Perirenal Adipose Tissue

PRAT, the AT surrounding the kidney, was previously assumed to merely mechanically support the kidneys. However, several studies postulated that not only PRAT has a pronounced role in regulating kidney function but is also associated with cardiometabolic risk factors. Clinical studies suggest that excess PRAT is associated with higher risk of CVDs (423, 424). The weight of PRAT has the highest partial correlation coefficient with CVDs among other AT (425). Indeed, excess PRAT is believed to contribute to the decrease in kidney function, regardless of obesity, in hypertensive patients (426). A recent study reviewed the possible mechanisms of PRAT in regulating CVDs including neural, humoral, and direct kidney related regulation (425). PRAT is shown to synthesize and secrete adipokines and several pro-inflammatory cytokines (425, 427). PRAT in pigs with obesity-related metabolic dysfunction showed elevated levels of pro-inflammatory macrophage infiltration and TNF-α expression (428). Moreover, excess PRAT secrets leptin which in turn activates MAPK pathway and further exacerbates renal vascular and endothelial damage (429). An interesting study in rats have shown that injecting Leptin directly into PRAT activated the adipose afferent reflex without changing the systemic sympathetic activity, indicating a direct regulation of cardiovascular function by PRAT (430). Interestingly a study on diabetic fatty rats found that the inhibition of PRAT inflammation, mainly inhibiting IL-6, IL-1b, and TNF-α, reduced renal inflammation and alleviated diabetic nephropathy (431). As such PRAT inflammation is assorted with adverse cardiometabolic risk factors and is a main predictor of CVD.

## The Adipose Immune System as o Regulator of Adaptive Thermogenesis

### UCP1-Dependent and Independent Thermogenesis

Uncoupling protein 1 (UCP1) is an inner mitochondrial membrane protein that uncouples oxidative phosphorylation from the production of ATP through a FA/H^+^ symport mechanism (2). UCP1 expression is mainly driven through β3-adrenoreceptors (β3-AR) stimulation by sympathetically and non-sympathetically produced norepinephrine in thermogenically active adipocytes. Although being the most efficient and qualitatively significant thermogenic effector, it was demonstrated that UCP1 is dispensable for cold-induced and diet-induced thermogenesis. Therefore, it was proposed that less-efficient thermogenic pathways downstream of β3-ARs also contribute to adaptive thermogenesis (432).

Creatine cycling, that is the phosphorylation of creatine by creatine kinase and its subsequent hydrolysis, participates in energy transfer from ATP-rich to ATP-poor cellular regions (2). Creatine futile cycling appears to occur in all fat depots and blocking creatine cycling promotes obesity in HFD-fed mice (2). Lipolysis/re-esterification cycling has also been proposed to mediate adaptive thermogenesis based on the ATP demand of triacylglycerol synthesis (2). This pathway proposes that adipocytes break down fat and subsequently re-esterifies FAs by way of glycerol 3-phosphate. Importantly, It was also shown that triglyceride/FA cycling is induced in WAT upon HFD feeding (433).

A role for calcium transport in non-shivering thermogenesis has also been proposed (2). Calcium sequestration in the sarcoplasmic (SR) and endoplasmic (ER) reticulum is mediated by the SR/ER calcium ATPase (SERCA) pump. SERCA activity in the AT is regulated by phospholamban (PLB) (434). Interestingly, it was shown that PLB is upregulated in UCP1-deficient beige fat with no difference in the expression of SERCA suggesting compensatory thermogenesis (435).

Finally, the UCP1-independent proton leak by the ubiquitously expressed inner membrane protein, mitochondrial ADP/ATP carrier (ACC), that is initiated at high membrane potential, contributes to adaptive thermogenesis (2).

### Adaptive Thermogenesis Across Adipose Depots

#### Brown Adipose Tissue

The first insights into the implication of BAT in thermogenesis and its contribution to energy expenditure started with the demonstration of a reduced GDP binding to BAT mitochondria of cold-exposed obese ob/ob mice relative to lean siblings (436). Then, Rothwell and Stock observed an increased sympathetic activity in BAT following overnutrition in rats (437). The identification of human BAT and the subsequent observations that a reduced BAT level induces obesity ignited investigation into BAT-mediated non-shivering thermogenesis. In comparison to WAT, which is more prone to inflammation than BAT (31), relatively little is known about the processes driving BAT chronic inflammation. However, increasing evidence suggests that BAT inflammation alters its thermogenic activity through the induction of IR (438, 439). Although mainly composed of brown adipocytes and their precursors, BAT also contains a variety of immune cells such as neutrophils, macrophages and lymphocytes (440, 441). Chronic inflammation of BAT was associated with a shift of BAT immune cells where M1 macrophages drive BAT whitening (442, 443).

#### Subcutaneous Adipose Tissue

Cold exposure and β_3_-AR stimulation induced the expression of UCP1 in scWAT of humans (444, 445). Nevertheless, despite the increased UCP1 expression in scWAT, cold acclimation was shown to reduce mitochondrial uncoupling-mediated fat oxidation in inguinal scWAT, while increasing the capacity to export FAs (446). Indeed, the consumption of HFD induced scWAT inflammatory and immune responses (447). These derangements were reversed by intermittent fasting, which increased the expression of UCP1, β_3_-ARs and adiponectin, while it attenuated the expression of pro-inflammatory and pro-apoptotic markers in scWAT (448). In an AMPK gain of function mutant mice, scWAT exhibited a morphological similarity to brown adipocytes with no detectable UCP1 expression but increased energy expenditure suggesting the activation of UCP1-independent thermogenesis (449). It was demonstrated that PPARγ agonism induced scWAT browning, while PPARγ deletion in inguinal scWAT inhibited thermogenesis and was associated with IR (450, 451).

#### Perivascular Adipose Tissue

The peculiarity of PVAT, being a hybrid AT and especially the resemblance of aortic PVAT to classical BAT in morphology and UCP1 expression, suggests that PVAT possesses a similar thermogenic potential (369). Indeed, it was shown that PVAT deletion resulted in a reduction of whole body temperature (452). The proximity of PVAT to the vascular wall suggests a possible implication of PVAT thermogenic processes on the pathophysiology of vascular diseases (33). We recently identified an increased expression of UCP1 in PVAT of HFD-fed rats, which was associated with localized PVAT inflammation contributing to MetS-associated vascular dysfunction (32). The targeting of PVAT UCP1 was also put forward as means to limit its detrimental effect on PVAT hypoxic predisposition (33). Such a proposition was made based on an assumed exaggerated oxygen consumption triggered by increased UCP1 expression and further complicated by the observed adipocyte hypertrophy in a combination of events less likely to occur in other adipose depots. However, increased UCP1 expression is typically viewed as beneficial where it serves as a route of energy assimilation that might be of value in diabetes and obesity. Yet, many of the tools shown to increase adipocyte glucose consumption and increased UCP1 expression *in vitro* failed to produce any effect when used *in vivo*, and even resulted in an opposite effect of decreased UCP1 expression (453, 454).

#### Epicardial Adipose Tissue

EpiCAT adipocytes express genes and secrete adipokines that are involved in thermogenesis (455). Adult human EpiCAT was shown to possess molecular features characteristic of beige adipocytes with relatively abundant expression of UCP1 (456). Opposite to findings in PVAT, an increased expression of UCP1 in EpiCAT was associated with a downregulation of ROS production and immune response (457, 458). Indeed, EpiCAT thermogenic activity was impaired in patients suffering from atrial fibrillation and heart failure with reduced ejection fraction (459, 460). Moreover, during the progression of atherosclerosis, EpiCAT was shown to undergo a phenotypic conversion from BAT to WAT, which further promoted the development of atherosclerosis (461). Nevertheless, exploiting EpiCAT browning for the treatment of CVDs remains controversial (33). As such, detailed examination of the role of thermogenesis modulation in PVAT and EpiCAT is required since both depots are particularly pertinent to the development of CVD in metabolic dysfunction.

#### Perirenal Adipose Tissue

Human PRAT has been shown to possess unilocular and multilocular UCP1^+^ adipocytes (462, 463). Indeed, several studies associated PRAT browning with aging and the female sex (464, 465). Also, bigger unilocular adipocytes with reduced UCP1 expression were detected in the PRAT of hypertensive patients (466).

#### Epidydimal Adipose Tissue

EpiWAT expresses UCP1 in rats age-dependently (467). The chronic agonism of PPARγ in EpiWAT promoted UCP1 expression and WAT browning (347). Indeed, the ectopic expression of very low levels of UCP1 in EpiWAT was shown to reverse IR in obese mice and epididymal beige adipocytes were shown to employ prominent creatine cycling (468, 469). Cold exposure improved metabolic dysfunction in obese mice through activating BAT thermogenesis and inducing EpiWAT browning (470). Moreover, cold-induced browning of VAT and improvement of insulin sensitivity were blunted following the knockdown of UCP1 in EpiWAT (471). Additionally, housing mice at room temperature induced EpiWAT thermogenesis, which was associated with a decreased M1 macrophage infiltration and improved insulin sensitivity (472). It was also shown that infused M2 macrophages in obese rats homed to EpiWAT reversing the M1 macrophage-dominant phenotype, enhancing UCP1 expression and ameliorating IR (473).

#### Mesenteric Adipose Tissue

It was demonstrated that β3-AR agonism in HFD-fed rats not only decreased the mass of WAT but also induced the appearance of multilocular, UCP1^+^ adipocytes in MAT (474, 475). These early observations indicated that MAT can be thermogenically induced. Indeed, cold exposure induced a sympathetic response in MAT of rats, evidenced by an increased level of tyrosine hydroxylase (476). Importantly, chronic cold exposure induces non-sympathetic catecholamine production leading to an increased level of NE in addition to the stimulation of M2 macrophage infiltration, pro-inflammatory cytokines reduction, and UCP1 induction (476, 477).

### Adipose Immune System and Adaptive Thermogenesis

#### Adipose Immune Cells and β_3_-AR Stimulation

**Figure 3:**
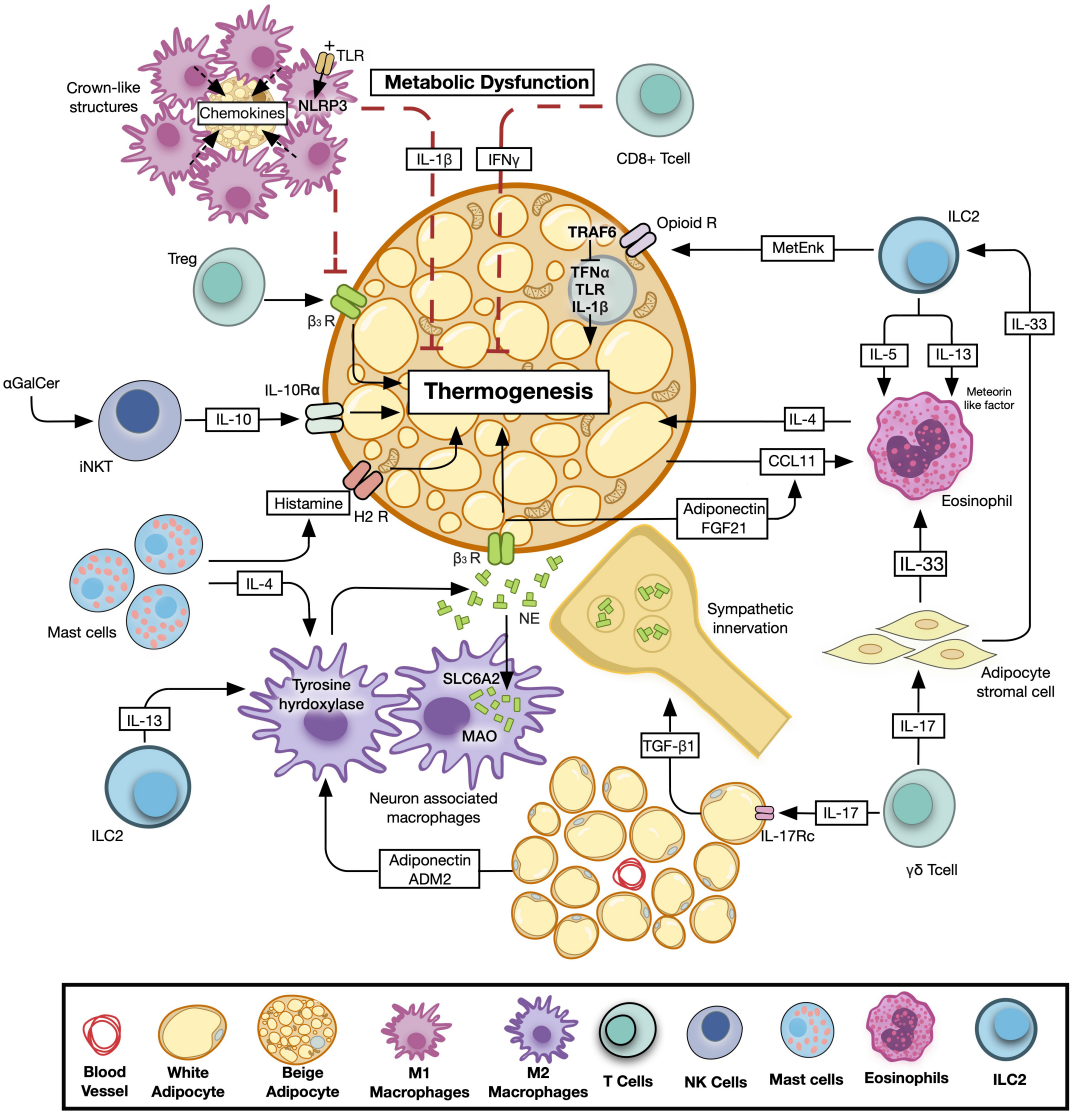
Immune cells-mediated regulation of adaptive thermogenesis. Different types of immune cells exert various modes of control on thermogenesis by either directly modulating the adipocyte function or affecting sympathetic nerve activity and norepinephrine turn-over. Pathways promoting thermogenesis are depicted in black, while inhibitory pathways are shown in red. ADM2, Adrenomedullin-1; β3-AR, Beta 3-adrenergic Receptor; CCL11, C-C motif chemokine 11; FGF21, Fibroblast Growth Factor 21; H2R, Histamine 2 Receptor; γGalCer, Alpha-galactosylceramide; IL, Interleukin; ILC, Innate Lymphoid Cell; MAO, Monoamine Oxidase; NE, Norepinephrine; Opioid R, Opioid Receptor; SCL6A2, Solute Carrier Family 6 Member 2; TGF-β, Transforming Growth Factor Beta; Treg, Regulatory T Lymphocyte.

β-AR stimulation is pivotal to the induction of thermogenesis. Sympathetically-released NE stimulates the release of adipokines and FGF21 from adipocytes, promoting PGC1α and UCP1 expression, oxidative metabolism, and mitochondrial biogenesis (478, 479). FGF21 also induces the release of CCL11 in murine scWAT, which promotes the recruitment of IL-4-secreting eosinophils and the proliferation of PDGFRα^+^ beige adipocytes in an IL-4Rα-dependent manner (480, 481). Moreover, eosinophils and ILC2s were shown to induce β_3_-AR signaling through IL-4/IL-13-dependent induction of tyrosine hydroxylase expression in ATMs, promoting the release of NE (215, 482). Also, the selective deletion of Mecp2 in BAT macrophages reduces UCP1 expression as a result of impaired innervation (441). Nevertheless, recent evidence suggests that ATMs are not likely to contribute to the induction of adaptive thermogenesis by directly producing NE (483). Sympathetic neuron-associated macrophages increased in HFD-fed mice AT and were recently shown to express the NE transporter, SLC6A2 and the NE degrader, monoamine oxidase (MAO), where the inhibition of SLC6A2 increased AT thermogenesis (484). Conversely, CLS-associated ATMs were shown to phagocytose white adipocytes and secrete chemokines that drive the recruitment of beige adipocyte precursors (263). Tregs were also shown to enhance β_3_-AR signaling in scWAT but not in VAT of female and to a lesser extent in male mice by suppressing M1 and inducing M2 macrophages (485). γδT cells were also shown to promote AT innervation by driving the expression of TGFβ1 in parenchymal cells via the IL-17 receptor C (IL-17RC), where the ablation of IL-17RC signaling pathway or γδT cells impaired sympathetic innervation and thermogenesis (486). The interaction among immune cells, adipocytes, and sympathetic nerve terminals is summarized in Figure 3.

#### Macrophages

M1 macrophages suppress the induction of thermogenic adipocytes in obese AT of mice (487). Conversely, adiponectin-induced M2 macrophages drive scWAT thermogenesis in cold-exposed mice and the depletion of either the macrophages or adiponectin reduces scWAT browning (488). The browning effect of adrenomedullin 2 (ADM2), a white adipocyte-produced factor that increases UCP1 expression, is also mediated by M2 macrophages (489).

The activation of pattern recognition receptors in AT-infiltrating macrophages was shown to suppress thermogenesis. LPS-activated TLR4 receptors of macrophages repressed β3-AR-induced adipocyte browning, caused mitochondrial dysfunction, and increased ROS production (490). Moreover, the activation of NLRP3 inflammasome in macrophages attenuated UCP1 induction in cultured adipocytes in an IL-1β-dependent manner (490). Furthermore, adipocyte-specific deletion of transforming growth factor-activated kinase 1 (TAK1) but not TNF receptor associated factor 6 (TRAF6), increased the expression of beige markers in WAT. TAK1 deletion in WAT increases AMPK phosphorylation, PGC-1α abundance, non-canonical NF-κB signaling, and markers of M2 macrophages while inhibiting canonical NF-κB signaling (491). Conversely, the deletion of TRAF1, an inhibitory adapter of TNFα, IL-1β, and TLRs enhanced leukocyte accumulation and potentiated the proinflammatory signaling of macrophages in HFD-fed mice (492). Nevertheless, TRAF1-deficient mice were protected from metabolic derangements and exhibited an improved IR partially by β3-AR-mediated induction of UCP1-dependent thermogenesis (492).

#### ILC2s

Activation of murine ILC2s with IL-33 induced the proliferation of beige adipocyte progenitors and increased WAT browning through an IL-4/IL-13-dependent pathway involving eosinophils (480). The recruitment of IL-4^+^ eosinophils was driven by ILC2-secreted IL-5 and IL-13. ILC2-produced BMP7 was also demonstrated to induced the differentiation of adipocyte progenitors into brown adipocytes (493). ILC2s also induce thermogenesis through the production of the opioid-like peptide methionine enkephalin (MetEnk) (316, 494). The stimulation of ILC2s with IL-33 induced the production of MetEnk that signaled through opioid receptors in scWAT and BAT to promote thermogenesis (316). Mice either treated with MetEnk or adoptive transfer of IL-33-activated ILC2s increased the expression of UCP1 in scWAT even in mice deficient in eosinophils or IL-4Rα demonstrating a direct activity of ILC2s on opioid receptors to induce thermogenesis (316).

#### γδT Cells

AT-resident γδT cells were recently shown to regulate body temperature through the production of IL-17A upon cold exposure, which regulated IL-33 production by adipose stromal cells (268). Mice deficient in γδT cells or IL-17A exhibited decreases in both ST2^+^ Tregs and IL-33 abundance in VAT and dysregulated core body temperature at thermoneutrality and upon cold exposure (268). Given the critical role of IL-33 in regulating insulin homeostasis and thermogenesis (495), IL-17-deficient mice were cold-intolerant (268). γδT cell-deficient mice also exhibited a reduced UCP1 expression and energy expenditure upon cold exposure (268).

#### iNKT Cells

The selective loss of IL-10Rα in adipocytes as well as the global depletion of IL-10 enhanced thermogenesis (496). Early reports demonstrated that the activation of adipose iNKT cells with αGalCer induced potent weight loss in obese mice (282, 284, 286, 497, 498). It was recently shown that iNKT cell-induced weight loss occur through the induction of FGF21-dependent adaptive thermogenesis. The intraperitoneal administration of αGalCer into obese mice induced a significant reduction of AT mass under thermoneutral conditions, which was accompanied by an increased WAT browning and energy expenditure (499). FGF21-deficient mice exhibited a blunted, but not fully ablated response toward αGalCer suggesting that iNKT cells drive thermogenesis through an FGF21-independent mechanism (499).

#### Mast Cells

BAT MC-released histamine is thought to play a β3-AR-independent role in thermogenesis through its interaction with H2 receptors (500). Upon cold exposure, MCs were recruited to WAT and exhibited an enhanced histamine degranulation in both lean and obese subjects, which was positively correlated with UCP1 expression and thermogenesis (501). Indeed, in response to cold, MCs also release IL-4 along with other factors driving UCP1 expression and WAT browning (502). Nevertheless, it was also proposed that MC deficiency in mice increases WAT browning by promoting adipocyte differentiation as MC-derived serotonin inhibited WAT browning (503). Nevertheless, these findings were based on a murine model in which c-kit tyrosine kinase is mutated and thus, a careful interpretation of the results is required. Furthermore, several other genetic models of MC depletion showed no association between MC function and obesity (228, 229).

#### T and B Lymphocytes

Several studies revealed a potential function of Tregs of scWAT and BAT in regulating thermogenic homeostasis. Systemic deletion of Tregs impaired oxygen consumption upon cold exposure (504). Additionally, the T cell-specific STAT6/PTEN axis is thought to mediate the link between β_3_-AR stimulation and Treg cell induction in both BAT and scWAT (505, 506). Indeed, UCP1-deficient mice exhibited reduced Tregs in BAT and scWAT (506). B and T lymphocytes were also shown to play a role in thermoregulation. Rag1-deficient HFD-fed mice, lacking both T and B lymphocytes, exhibited decreased UCP1 expression (507). Conversely, deleting Rag1 in lean mice housed at room temperature resulted in an increased UCP1 expression and energy expenditure (508). Moreover, a decreased CD8^+^ but not CD4^+^ T cells is believed to contribute to adipocyte browning mainly due to a decreased IFN-γ secretion (508).

#### Eosinophils

Eosinophil-derived IL-4 drives M2 macrophage polarization, promoting the secretion of catecholamines that drive WAT browning (215). As mentioned before, the role of M2 macrophages in local catecholamine production has been questioned. Nevertheless, this does not preclude the implication of eosinophil-derived catecholamines in WAT browning. Indeed, PVAT eosinophils were shown to promote PVAT browning by locally producing catecholamines (509). Moreover, mice lacking eosinophils exhibited an impaired thermogenic capacity of scWAT following cold exposure (215). Meteorin-like is another factor linking eosinophils to WAT browning, where it was shown to stimulate IL-4 secretion from eosinophils and macrophage M2 polarization in AT following cold exposure (510). In addition to ILC2-derived IL-33-dependent eosinophils recruitment to AT, IL-33 was also shown to recruit eosinophils in the absence of ILC2s (178). It was recently demonstrated that the transcriptional repressor krüppel-like factor 3 (KLF3)-deficient mice exhibited profound WAT beiging, which was accompanied by an accumulation of AT eosinophils (511).

## Immunomodulating Adipose Tissue Inflammation in Metabolic Disorders and Cardiovascular Diseases

Strategies to modulate AT inflammation are multi-faceted, they include physical exercise, lifestyle modifications, in addition to several pharmacological and non-pharmacological interventions. Likewise, treatment of CVDs focuses on similar strategies that impact AT. In this section, we will tackle the contribution of different modalities on AT inflammation in CVDs.

### Exercise and Lifestyle Modifications

One of the first strategies to decrease the severity and complications of CVDs is to limit food intake and increase energy expenditure, this is mainly due to the fact that most patients with CVDs are overweight or obese. Exercise and lifestyle modifications lower the mortality risk, improve quality of life and have been extensively studied. Physical activity improves insulin sensitivity and alters AT adipokine expression, which affect whole-body metabolic health in human subjects (512, 513). Recent studies highlighted the mechanistic pathways, linking those two interventions with decreasing AT inflammation. The protective effects of regular physical activity is accompanied with reduction of visceral fat mass along with an anti-inflammatory pathway (514). Physical exercise exerts a direct anti-inflammatory effect by inducing an acute elevation in IL-6 and IL-10 and an inhibition of TNF-α (515). The anti-inflammatory effect of exercise also comprises the inhibition of macrophage infiltration and the induction of ATMs phenotypic switch toward the M2 phenotype in obese mice (515, 516). Zielger et al. demonstrated that exercise enhances the anti-inflammatory phenotype in VATs of old mice (517). Endurance training regardless of weight loss induced an increase in M2 macrophages in scWAT (513, 518). The role of physical activity in thermogenesis and WAT browning is debated as some reported scWAT browning with bicycle training programs while another study failed to find a correlation between aerobic exercises and recruitment of beige adipocytes (519, 520). Nevertheless, more studies should target the exact role of AT remodeling following physical activity. Indeed, long-term anti-inflammatory effects of chronic physical activity could be required for a pronounced AT remodeling required to decrease CVD risks.

### Diet Modification and Weight Loss

#### Fasting

Several fasting regimens were introduced as an alternative or a complementary intervention to restricted caloric diets in improving cardiometabolic endpoints in related diseases (521, 522). Indeed, several studies on experimental animals and recent human investigations highlighted the importance of fasting in metabolic activity regulation, blood pressure, and atherosclerosis reduction, as well as health optimization (522–524). The most common types of fasting regimens include intermittent fasting (IF), periodic fasting (PF), short term fasting, and religious fasting. IF has a crucial role in adaptive cellular responses being able to reduce inflammation, oxidative stress, optimize energy metabolism, and cellular bioenergetics (524). Fasting is an effective strategy for improving cardiometabolic profile in cases of IR, stroke, prediabetes, and diabetes (525, 526). Moreover, IF modulates the susceptibility of inflammatory diseases by decreasing peripheral monocyte pools and modifying their metabolic activity through AMPK and PPARα pathways (527). On the AT level, fasting enhances mitochondrial biogenesis in visceral adipocytes. Short term fasting suppressed thermogenesis in inguinal WAT and iBAT in a mouse model (528). Another fasting regimen, every other day fasting, was shown to induce beiging of WAT thus reversing HFD-induced obesity and associated metabolic disorders in mice (529). The metabolic effects of IF are largely mediated by adipose thermogenesis; fasting-induced adipose VEGF, which is thought to act on eosinophils, Th2, and ILC2 to promote M2 polarization was linked to WAT browning (530). Moreover, fasting induces a reduction of IL-1 and IL-6 in VAT and scWAT and IL-6 in intraperitoneal WAT (531). Moreover, biomarkers of inflammation in EpiWAT and BAT were reduced in mice following IF. The latter study presented IF as a preventive and therapeutic intervention to protect mice against MetS and obesity (532). On the other hand, fasting reduces leptin levels triggering a profound metabolic state as well as regulating T lymphocytes and cytokine production in obese animal models and human trials (533–535). In addition to that, a new randomized control trial has also linked fasting to reduction of leptin levels (536).

#### Dietary Modifications

Dietary modification is the cornerstone in preventing cardiometabolic diseases. Weight loss approaches as well as initiating certain diet regimens lower CVD events significantly and reduce mortality (537). Reports correlating dietary manipulation, such as in high protein diets, phosphate diet, and ketogenic diet, to AT inflammation suggest that certain diet regimens can play a critical role in modulating cardiometabolic diseases.

Long term intake of high protein diet in obesity-prone rats reduced food intake and WAT mass while improving basal blood sugar, insulin levels, leptin, and triglyceride levels in addition to glucose tolerance (538). On the other hand, a clinical study suggested that phosphorus supplementation is involved in modulating glucose and insulin serum levels (539). Another study reported that high dietary intake of phosphate in rats can influence lipid and glucose metabolism by upregulating lipolytic gene expression and reducing WAT accumulation (540).

Ketogenic diet (KD), which consists mainly of ingesting healthy fats, improved long-term blood glucose control and subsequently decreased the use of anti-diabetic agents in human studies (541, 542). KD also improved the CVD biomarkers in T2DM patients (543). Moreover, short term feeding of KD was shown to modulate AT immune cells, where it reduced macrophage infiltration and the expansion of γδ T cells in VAT (279).

Mediterranean Diet (MD) is composed of a balanced combination of fruits, vegetables, fibers, fish, poly saturated fats as well as low intake of meat and dairy products in addition to moderate intake of red wine (544). The adherence to MD is known to protect humans against CVDs, MetS, onset of various types of cancer, and aging (545, 546). Several studies documented that certain typical food of the MD including olive oil, tomato, and red wine induce anti-inflammatory properties and could be even insulin-sensitizing (547, 548). For example, tomato juice mitigates AT inflammation; a 20-days duration of consumption could decrease TNF-α, IL-6, and IL-8 (549, 550). Moreover, tomato juice supplementation could reduce body weight, blood cholesterol levels as well MCP-1 (551).

### Anti-diabetic Drugs

#### Metformin

Besides being widely used for DM2 treatment, Metformin reduces CVD risk, induces weight loss, and improves insulin sensitivity (552). Metformin has been proposed to reduce adipocyte stores and initiate a metabolically healthy adipocytes distribution (553). The beneficial effects of metformin also include reducing visceral AT, a mechanism that is thought to be related to FA oxidation and an upregulation of adaptive thermogenesis (554). Emerging body of evidence, including work done by our team, documented that metformin can exhibit immunomodulatory features, an anti-inflammatory effect that is shown to be independent on glycemic control (32, 555, 556). Metformin activates the anti-inflammatory macrophage polarization; it lowers the pro-inflammatory cytokine production through elevating M2 macrophage and lowering M1 macrophages (557).

Metformin also decreases MCP-1 in isolated human AT cultures, suggesting an improved low-grade inflammation (558). Moreover, metformin has been shown to alter CRP, NF-κB expression in addition to reducing advanced glycation end products (559–561). All in all, the anti-inflammatory effects of metformin and its ability to reduce several inflammatory related illnesses is becoming more apparent (552).

#### Thiazolidinediones (TZDs)

Besides the use of TZDs in T2DM, a beneficial role of Pioglitazone lies in reducing cardiovascular events (562). In fact, TZDs activation of PPARγ not only enhances adipogenesis but also reduces fat deposition in tissues, and attenuates the inflammatory cytokines release in obesity (563, 564). Moreover, TZDs repress NF-kB thus restore M2 macrophage phenotype, and prompt the recruitment of regulatory T cells in AT (263, 565, 566). Therefore, this reveals a possible involvement of TZDs in an immunomodulatory mechanism in AT that may benefit patients with CVDs, yet the exact definitive pathway has not been established. Expanding on the beneficial effects of PPARγ agonism in limiting AT chronic low-grade inflammation in metabolic disorders, glitazones-like, multi-targeting drug ligands (MTDLs) were rationally designed (567). Importantly, these drugs were partial PPARγ agonists, potent COX-2 antagonists and moderate 15-LOX inhibitors. This balanced modulation of the three inflammatory targets allows for a more effective targeting of AT inflammation and possibly limit its cardiovascular complications (568).

#### Glucagon-Like Peptide-1 (GLP-1) Receptor Agonists

Several GLP-1 receptor agonists, as Liraglutide, have been developed to mimic the glucose-lowering and anorexic effects of Glucagon-like peptide-1 (GLP-1) to treat obesity and T2DM. As AT express GLP-1, Liraglutide has been effective in controlling glucose levels, promoting weight loss, and reducing total adiposity (569–571). In addition, in a clinical trial, Liraglutide has been shown to decrease the risk of myocardial infarction in patients with T2DM and high CVD risk (572). More studies on the effect of GLP-1 agonists documented their protective roles against endothelial cell dysfunction, and therefore atherosclerosis by reducing CRP and plasma lipids (573, 574). As such, GLP-1 agonists could provide protection against CVDs through AT mass reduction and inflammation.

#### Sodium-Glucose Cotransporter (SGLT2) Inhibitors

Similar to GLP-1 receptor agonists, SGLT-2 inhibitors are shown to reduce blood glucose levels and CVDs risk and mortality (575, 576). Treatment with Empagliflozin, an SGLT-2 inhibitor, has been shown to induce weight loss when given in combination with other anti-diabetic medication (577, 578). In obese mice, Empagliflozin was shown to promote utilization and browning of AT as well as reduction of IR and inflammation, a pathway linked to M2 macrophage polarization (579). As such, beside the anti-diabetic effects of SGLT-2 inhibitors, strong evidence appears in their effect on AT remodeling and anti-inflammatory pathway; yet the exact mechanism is to be elucidated.

### Surgical Interventions

Bariatric surgery is the most effective treatment option in obese patients for weight loss whether due to food restriction, malabsorption, or both (580). Following the surgery and independent on weight loss; IR, CVDs, and mortality rates are all reduced (537, 581, 582). Importantly, it is expected that a late phase reduction of AT inflammation could be in favor of all the metabolic consequences of the surgery (583). However, when looking back to literature, contradictory results are revealed. Some have documented a decrease in inflammatory mediators of AT after the surgery-induced weight loss, and others have reported no further change (584–587). Reports generally assess subcutaneous AT depot, as it is easier in sampling. However, more studies should be warned to confirm or elucidate the effects of bariatric surgery on AT inflammation. Sampling from visceral AT and other sites should be done as it is more prone to inflammatory changes.

## New Avenues for Adipose Tissue Immunomodulation in Metabolic Disorders and Cardiovascular Diseases

AT inflammation is associated with an increased production of pro-inflammatory cytokines including IL-1β, TNF-α, IL-6, and IFN-γ. Anti-inflammatory treatments were proposed to contribute to the treatment of diabetes and its vascular complications (588). The antagonism of IL-1R improved IR in T1D patients and DIO mice (589, 590), and in patients with impaired glucose tolerance (591). One study however highlighted the importance of combining IL-1R antagonism treatment with proper dieting for the treatment of obesity (592). Moreover, inhibiting TNF-α in psoriasis patients with MetS decreased macrophage infiltration and pro-inflammatory cytokines levels in umbilical fat (593). Interestingly, a combined inhibition of IL-1β and TNF-α was more effective in improving IR in T2D rats (594). Similarly targeting IL-6 improved IR and normalized adipokine levels in MetS and fructose-fed rats (595, 596). Nevertheless, this mechanistic link was not evident in clinical trials (597). Indeed, IL-6 was shown to drive exercise-induced weight loss in subjects with visceral obesity (598). The complexity of the AT immune landscape driving AT inflammation in the MetS and the response of these cells to the various pro-inflammatory cytokines dictate the efficacy of these approaches. However, simply targeting pro-inflammatory cytokines with either receptor inhibitors or monoclonal antibodies for the treatment of metabolic and cardiovascular diseases is not yet a valid therapeutic strategy and requires further investigation.

The metabolic reprogramming in response to nutritional excess and scarcity of the various immune cells is not universal. Indeed, metabolic modulation emerged as a novel concept in cancer immunotherapy (599). Th2 immunity in AT supports metabolic health and thus, targeting Th2 immunometabolism represents a valuable therapeutic strategy in metabolic disorders. Several reports on immunometabolism-targeted treatments in cancer and autoimmunity can be repurposed for metabolic diseases. For example, in a model of allograft rejection, blocking glycolysis and glutamine metabolism inhibited CD4^+^ and CD8^+^ T cell induction and promoted the generation of allospecific Treg cells (600). Indeed, expanding AT Treg cells hold much promise for the treatment of metabolic disorders and their cardiovascular complications. Additionally, nanoparticles, liposomes, and glucan-shells carrying siRNA or specific drugs can be engineered to tissue-specifically target specific immune cells as exemplified by ATMs (601). Nevertheless, a profound knowledge of metabolic profile shifts of immune cells within the AT is still lacking and thus, direct interpretations of such shifts in other tissues cannot be extrapolated, particularly in the complex, dynamically-evolving AT. Emerging technologies such as RNA sequencing, metabolomics, proteomics and phage display will surely allow for the identification of novel peptide targets (602). Moreover, adaptive immunity and the acquisition of memory T cells in HFD-fed mice suggests an effect on subsequent episodes of weight gain following weight loss (603, 604). Modulating T cell memory has been achieved by targeting antigen presentation in conventional and non-conventional APCs (271) and checkpoint co-inhibitory interactions (605). Finally, emerging evidence indicates that immunometabolism is controlled epigenetically and through miRNAs, which affects cellular differentiation and polarization (606, 607). Nevertheless, further research is required to determine how metabolic dysfunction drives alterations in epigenetic histone modifications and how miRNAs affects the AT immune profile.

Accumulating evidence suggests a role for the gut microbiota in modulating metabolic homeostasis. Indeed, it was shown that the adoptive transfer of Th 17 cells contributed to metabolic homeostasis through an IL-17-dependent microbiota development (608). Moreover, it was demonstrated that the M2 macrophage-mediated helminth-associated Th2/Treg responses induce alterations in microbiota composition which was accompanied by protection against obesity (609, 610).

We have highlighted a key role for physical activity, different fasting regimens, and dietary modifications in limiting AT inflammation and IR. Nevertheless, their implications on different adipose depots, especially those of cardiovascular interest are not well-characterized. In fact, a depot-specific metabolic profiling is pivotal to delineate their differential effects. Moreover, anti-diabetic drugs and surgical procedures showed a favorable outcome on metabolic parameters and CVDs. In fact, these approaches reduced AT inflammation through mechanisms being revealed only recently. Therefore, it is pivotal that AT immune profiles in different depots be characterized following the administration of anti-diabetic drugs.

Finally, the induction of BAT activity and WAT browning has been proposed as a mean to curb obesity and combat CVDs. Indeed, the induction of different AT depots browning resulted in either favorable or detrimental outcomes. Targeting UCP1 was even proposed as the induction of browning in PVAT and EpiCAT was supposed to deteriorate vascular and cardiac functionality (33). Indeed, this strategy is still debated as clinical trials have not shown a significant improvement of metabolic parameters following the induction of thermogenesis (33). In addition to the non-selective impact on all adipose depots, the available UCP1 inhibitors possess a fairly high IC_50_ value (~20 μM) (611) precluding systemic administration without significant off-target and adverse effects. As such, immune modulation of thermogenesis might constitute a lucrative target for depot-specific intervention. As different depots possess variable intrinsic brown-like character, it is pivotal to further characterize this phenotype, the manner by which it is affected by the immune system in states of health and disease, and how increased energy expenditure leads to clinical significance. Significantly, the relative impact of the activation of different thermogenic pathways on AT inflammation in various adipose depots requires systematic examination. Whether the selection of one pathway over the other modulates activity and/or recruitment of disparate immune cells remains unknown. As well, the ability of a specific immune cell product/function to favor one pathway over the other has not been investigated.

## Conclusion

Metabolic and Cardiovascular diseases are multifactorial disorders to which contributes the inflammation of the AT. Several pharmacological and non-pharmacological interventions have been shown to exert their positive effects in these diseases, at least in part by modulating AT inflammation. Accumulating evidence implicates different immune cells in the regulation of AT inflammation and its consequences including IR. The metabolic reprogramming of AT immune cells and the alteration of the AT immune landscape are believed to drive AT inflammation, BAT thermogenesis and WAT browning. Further investigation is required to delineate the exact role of different immune cells and the consequences of their metabolic profile alteration in different adipose depots inflammation. A better comprehension of the mechanisms driving AT inflammation allows for the emergence of novel therapeutic strategies aimed at immunomodulating the AT.

## Author Contributions

IA, SH, HA-K, and AG participated in literature review and screening and contributed to manuscript writing. IA wrote the first draft of the manuscript. AE helped in overseeing and coordinating the work and participated in manuscript draft review. AE-Y developed the idea, supervised the work, reviewed and modified manuscript draft, and provided research funding support. All authors contributed to the article and approved the submitted version.

## Conflict of Interest

The authors declare that the research was conducted in the absence of any commercial or financial relationships that could be construed as a potential conflict of interest.
